# Immune checkpoint inhibitor–related myositis, myocarditis, and myasthenia gravis overlap syndrome: a systematic review and pooled analysis of individual cases

**DOI:** 10.3389/fimmu.2026.1854723

**Published:** 2026-07-29

**Authors:** Miao Wei, Tiantian Hu, Lili Yang, Yan Duan, Yifan Li

**Affiliations:** 1 Department of Intensive Care Unit , Shanxi Province Cancer Hospital / Shanxi Hospital Affiliated to Cancer Hospital, Chinese Academy of Medical Sciences / Cancer Hospital Affiliated to Shanxi Medical University, Taiyuan, China; 2 Department of Hepatobiliary and Pancreatic Surgery , Shanxi Province Cancer Hospital / Shanxi Hospital Affiliated to Cancer Hospital, Chinese Academy of Medical Sciences / Cancer Hospital Affiliated to Shanxi Medical University, Taiyuan, China

**Keywords:** immune checkpoint inhibitors, in-hospital mortality, myasthenia gravis, myocarditis, myositis, overlap syndrome

## Abstract

**Background:**

Immune checkpoint inhibitor (ICI)–related overlap syndrome involving myositis, myocarditis, and myasthenia gravis (3M overlap syndrome) is rare but potentially fatal. Current evidence is derived mainly from case reports and small case series, and individual case-level data on ancillary diagnostic findings, baseline comorbidities, treatment patterns, and short-term outcomes remain limited.

**Methods:**

We performed a systematic review of published case reports and case series and a pooled analysis of individual case data on ICI-related 3M overlap syndrome, supplemented by three patients from our center. The primary endpoint was all-cause in-hospital mortality. Univariable and multivariable logistic regression were used to explore factors associated with in-hospital mortality, and exploratory symptom correlation and hierarchical clustering analyses were performed to describe symptom co-occurrence patterns.

**Results:**

Ninety-five published reports comprising 133 patients were included, together with 3 additional institutional cases, for a total of 136 patients. The mean age was 69.8 ± 11.2 years, and 96/136 (70.6%) were male. Melanoma was the most common underlying malignancy (40/136, 29.4%). The median time from first ICI exposure to symptom onset was 22.0 days (IQR, 16.0–31.0).In-hospital outcome was ascertainable for all 136 patients; 53 (39.0%) died during hospitalization and 83 (61.0%) survived to discharge or transfer. Most patients received systemic corticosteroids (133/136, 97.8%) and second-line immunomodulatory therapy (119/136, 87.5%). In the pre-specified multivariable analysis (age, fatigue, and complete conduction block), older age (adjusted odds ratio [aOR], 1.11 per year; 95% confidence interval [CI], 1.05–1.17; P < 0.001) and complete conduction block (aOR, 2.84; 95% CI, 1.10–7.34; P = 0.031) remained independently associated with in-hospital mortality, whereas fatigue was no longer statistically significant after adjustment (aOR, 1.85; 95% CI, 0.75–4.56; P = 0.181). Exploratory clustering identified a possible bulbar/axial symptom cluster characterized by dysphagia, dysarthria, and neck muscle weakness.

**Conclusions:**

ICI-related 3M overlap syndrome is an early-onset, fulminant immune-related adverse event with substantial in-hospital mortality. Older age and complete conduction block were factors associated with poorer in-hospital outcomes in this exploratory analysis; these findings should be regarded as hypothesis-generating and require validation in prospective registries or multicenter cohorts.

## Introduction

The widespread use of immune checkpoint inhibitors (ICIs) has substantially improved survival across multiple malignancies, but has also led to a broad spectrum of immune-related adverse events (irAEs), some of which are life-threatening (1, 2). Among these toxicities, ICI-related myocarditis is uncommon but is characterized by abrupt onset, rapid progression, and high fatality (3, 4). ICI-related myositis is regarded as one of the earliest and most potentially fatal rheumatic and musculoskeletal irAEs (5), whereas ICI-related myasthenia gravis (MG) is more likely than classic MG to present with respiratory failure and death (6). Importantly, these entities are not always isolated. Concurrent involvement of the myocardium, skeletal muscle, and neuromuscular junction may occur, forming a highly fulminant overlap syndrome of myositis, myocarditis, and MG, hereafter referred to as the 3M overlap syndrome (7).

Recognition of ICI-related 3M overlap syndrome has increased in recent years, but the available evidence remains derived largely from case reports, small case series, and descriptive reviews. Previous systematic reviews have suggested that the syndrome occurs predominantly in older men, tends to develop early after ICI initiation, is most frequently reported in patients with melanoma, and carries a high mortality rate (8, 9). Nevertheless, several gaps remain. First, earlier studies have focused mainly on descriptive summaries of clinical manifestations and treatment courses, whereas ancillary diagnostic findings, baseline comorbidities, concomitant non-ICI anticancer therapy, treatment patterns, and short-term in-hospital outcomes have not been systematically evaluated. Second, few reviews have examined factors associated with in-hospital mortality at the individual case level, limiting clinically useful clues for early risk stratification. Third, follow-up duration and outcome reporting vary considerably across published cases, making longer-term endpoints—particularly 28-day mortality—difficult to compare consistently. By contrast, in-hospital mortality is more consistently available and may better reflect the true severity of the acute phase of this syndrome.

Against this background, we conducted a systematic review of case reports and case series and a pooled analysis of individual case data on ICI-related 3M overlap syndrome, incorporating three additional patients from our center. We aimed to summarize the epidemiologic profile, clinical manifestations, ancillary diagnostic findings (myocarditis evidence, myositis evidence, and MG/neuromuscular-junction evidence), baseline comorbidities, concomitant non-ICI anticancer therapy, treatment strategies, and in-hospital outcomes of this syndrome, and to explore clinical factors associated with in-hospital mortality. By leveraging a larger body of case-level evidence, we sought to provide more targeted evidence for early recognition, risk assessment, and clinical management.

## Methods

### Study design and registration

This study was a systematic review of published case reports and case series with a pooled analysis of individual case data from patients with ICI-related myositis, myocarditis, and MG overlap syndrome, supplemented by three patients from our center. This study was registered with PROSPERO (CRD420261331490).

### Search strategy

We systematically searched PubMed, Embase, the Cochrane Library, China National Knowledge Infrastructure (CNKI), and Wanfang Data for studies published from January 1, 2010, to April 1, 2026. Reference lists of the included studies and Google Scholar were also screened to identify additional reports. The search strategy combined controlled vocabulary and free-text terms related to ICIs, myocarditis, myositis, MG, overlap syndrome, case reports, and case series. Detailed search strategies for the core databases are provided in Supplementary Appendix 1, and the search terms were adapted as appropriate for the remaining databases.

### Eligibility criteria

Studies were eligible if they: (1) reported patients exposed to at least one ICI; (2) described concurrent or sequential occurrence of ICI-related myocarditis, myositis, and MG consistent with the 3M overlap syndrome; (3) were published case reports or case series, including the three patients treated at our center; (4) provided extractable individual-level data on demographics, clinical manifestations, investigations, treatment, and/or hospitalization outcomes sufficient for the pooled analysis; and (5) were published in English or Chinese.

We excluded: (1) cohort studies, pharmacovigilance studies, reviews, meta-analyses, commentaries, letters, and conference abstracts without extractable individual case data; (2) duplicate publications or overlapping datasets; and (3) cases with insufficient diagnostic evidence or missing key outcome data that precluded inclusion in the primary analysis. During revision, all included reports were re-examined and two cases were identified as duplicate publications of the same patients and were removed, yielding the final cohort of 133 unique published cases plus 3 institutional cases (n = 136).

### Study selection and data extraction

Two investigators independently screened the records according to the predefined search strategy. After title and abstract screening, potentially eligible reports underwent full-text assessment. Disagreements were resolved by discussion or adjudication by a third investigator. Data were extracted using a standardized form that captured:(1) demographic and oncologic data (age, sex, malignancy type, ICI agent and regimen, ICI cycles, and time from first ICI exposure to symptom onset); (2) baseline comorbidities, with pre-specified extraction of hypertension, diabetes mellitus, chronic kidney disease, coronary artery disease/ischemic heart disease, and obesity, together with other reported conditions; (3) concomitant non-ICI anticancer therapy administered with the ICI regimen at the index event, categorized as chemotherapy, chemoradiotherapy, or targeted/anti-VEGF/tyrosine kinase inhibitor therapy (dual ICI regimens were not counted as concomitant non-ICI therapy); (4) presenting symptoms and signs; (5) ancillary diagnostic findings, recorded separately as myocarditis evidence (cardiac biomarkers, ECG, echocardiography, cardiac MRI, endomyocardial biopsy, and autopsy), myositis evidence (CK or aldolase, EMG/NCS, muscle MRI, and muscle biopsy), and myasthenia gravis/neuromuscular-junction (MG/NMJ) evidence (acetylcholine receptor and other relevant antibodies, repetitive nerve stimulation, and edrophonium/ice-pack testing); (6) treatments, including systemic corticosteroids (with explicit recording of pulse-dose use), IVIG, PLEX, other immunosuppressive or targeted immunomodulatory agents, MG-directed symptomatic therapy, and supportive care; (7) length of hospital stay and time from symptom onset to initiation of irAE-directed treatment, when explicitly reported; and (8) clinical outcomes, with a focus on all-cause in-hospital mortality and patient-level disposition.

A finding was coded as present only when explicitly reported, as absent when explicitly denied or normal, and as “not reported” when the source did not address it; investigations or comorbidities not reported in the source publication were not assumed to be negative or absent, and denominators therefore vary across variables according to information available. Clinical data for the three institutional patients were collected retrospectively from the electronic medical record after approval by the ethics committee.

### Diagnostic criteria and reclassification

Two investigators independently reclassified the diagnoses of myocarditis, myositis, and MG using predefined criteria derived from the published literature (5, 10, 11). In brief, ICI-related myocarditis was diagnosed on the basis of compatible clinical features plus evidence of myocardial injury and supportive ECG, echocardiographic, cardiac magnetic resonance, endomyocardial biopsy, or autopsy findings after exclusion of alternative causes (10). ICI-related myositis was defined by compatible symptoms together with elevated CK or aldolase and supportive electromyography, skeletal muscle magnetic resonance imaging, or muscle biopsy findings (5). ICI-related MG was defined by compatible symptoms and/or signs together with positive MG-associated autoantibodies, positive pharmacologic or ice-pack testing, or abnormal electrophysiology (11). Any discrepancies in reclassification were resolved by consensus.

### Treatment and supportive care definitions

Treatment and supportive care definitions. Immunomodulatory treatment was categorized as systemic corticosteroids (including pulse-dose methylprednisolone, defined as ≥500 mg/day for at least three consecutive days) and second-line immunomodulatory therapy, comprising IVIG, PLEX, and additional immunosuppressive or targeted immunomodulatory agents. Because the timing of second-line therapy was inconsistently or incompletely reported, upfront versus stepwise escalation could not be reliably distinguished, and second-line use was therefore summarized descriptively. Mechanical circulatory support was defined *a priori* as venoarterial extracorporeal membrane oxygenation (VA-ECMO), Impella, intra-aortic balloon pump (IABP), or ventricular assist device; pacemaker or ICD implantation and continuous renal replacement therapy or dialysis were not considered mechanical circulatory support and were tabulated separately. Vasoactive or inotropic therapy and antiarrhythmic drugs were inconsistently reported and could not be pooled quantitatively.

### Risk of bias assessment

Methodological quality was assessed using the Joanna Briggs Institute critical appraisal tools for case reports and case series. The assessment focused on the reporting of patient demographics, diagnostic ascertainment, interventions, and outcomes. Study quality was appraised descriptively and was not used as an exclusion criterion.

### Statistical analysis

All analyses were conducted using R version 4.2.2 (http://www.R-project.org, R Foundation) and Free Statistics software (version 2.0). Continuous variables are presented as mean ± standard deviation or median (interquartile range [IQR]), as appropriate according to their distribution. Categorical variables are reported as n/N (%), using the number of patients with available data for each variable as the denominator. A statistical P-value below 0.05 was considered statistically significant.

The primary endpoint was all-cause in-hospital mortality. Univariable logistic regression assessed the association of clinical, diagnostic, and treatment-related variables with in-hospital mortality (**Supplementary Table 4**). Given the case-level nature of the source data, the variable completeness of reporting, and multiple comparisons, all univariable and multivariable analyses are interpreted as exploratory and hypothesis-generating; throughout the manuscript these results are described as “factors associated with in-hospital mortality” or “exploratory mortality-associated factors” rather than as predictors.

The pre-specified multivariable logistic regression model (**Table 1**) included age (per year), fatigue, and complete conduction block. Mechanical ventilation was excluded from the main multivariable model because it likely represents a downstream marker of disease severity on the causal pathway between severe 3M overlap syndrome and death; adjusting for it would risk overcontrol bias. Second-line immunomodulatory therapy was excluded because treatment exposure is time-dependent and is subject to survival-time and treatment-selection bias in retrospective case-level data. Complete-case analysis was used; complete conduction block was analyzed only among patients with available ECG information, and unreported ECG findings were not assumed to be normal. Missing data were not imputed. To further explore symptom co-occurrence patterns, we performed pairwise correlation analyses of commonly reported symptoms and visualized the symptom correlation matrix using hierarchical clustering. Given the heterogeneity and missingness inherent to case reports, this analysis was considered exploratory and was not intended for causal inference or formal subtype definition. For exploratory symptom analyses, pairwise associations among binary symptom variables were quantified using phi coefficients (equivalent to Pearson correlations for binary variables coded as 0/1) based on available case-level data, and hierarchical clustering was performed on the correlation matrix using Ward’s method.

**Table 1:** Primary and sensitivity multivariable logistic regression models for factors associated with in-hospital mortality.

Variable	Adjusted OR	95% CI	P value
Primary multivariable model			
Age (per 1-year increase)	1.11	1.05–1.17	<0.001
Fatigue	1.85	0.75-4.56	0.181
Complete conduction block	2.84	1.1-7.34	0.031
Sensitivity model			
Age (per 1-year increase)	1.11	1.05–1.17	<0.001
Dyspnea plus fatigue	2.37	0.69-8.07	0.168
Complete conduction block	2.72	1.07-6.94	0.031

OR indicates odds ratio; CI, confidence interval.

In the sensitivity model, dyspnea plus fatigue was entered in place of fatigue to assess the robustness of the association while avoiding collinearity or conceptual overlap.

### Ethics

This study was primarily a systematic review and did not involve direct patient intervention. Collection of the three institutional cases was approved by the hospital ethics committee, which waived the requirement for informed consent (approval number: KY2025024). The study was conducted in accordance with the Declaration of Helsinki.

## Results

### Study selection

**Figure 1:**
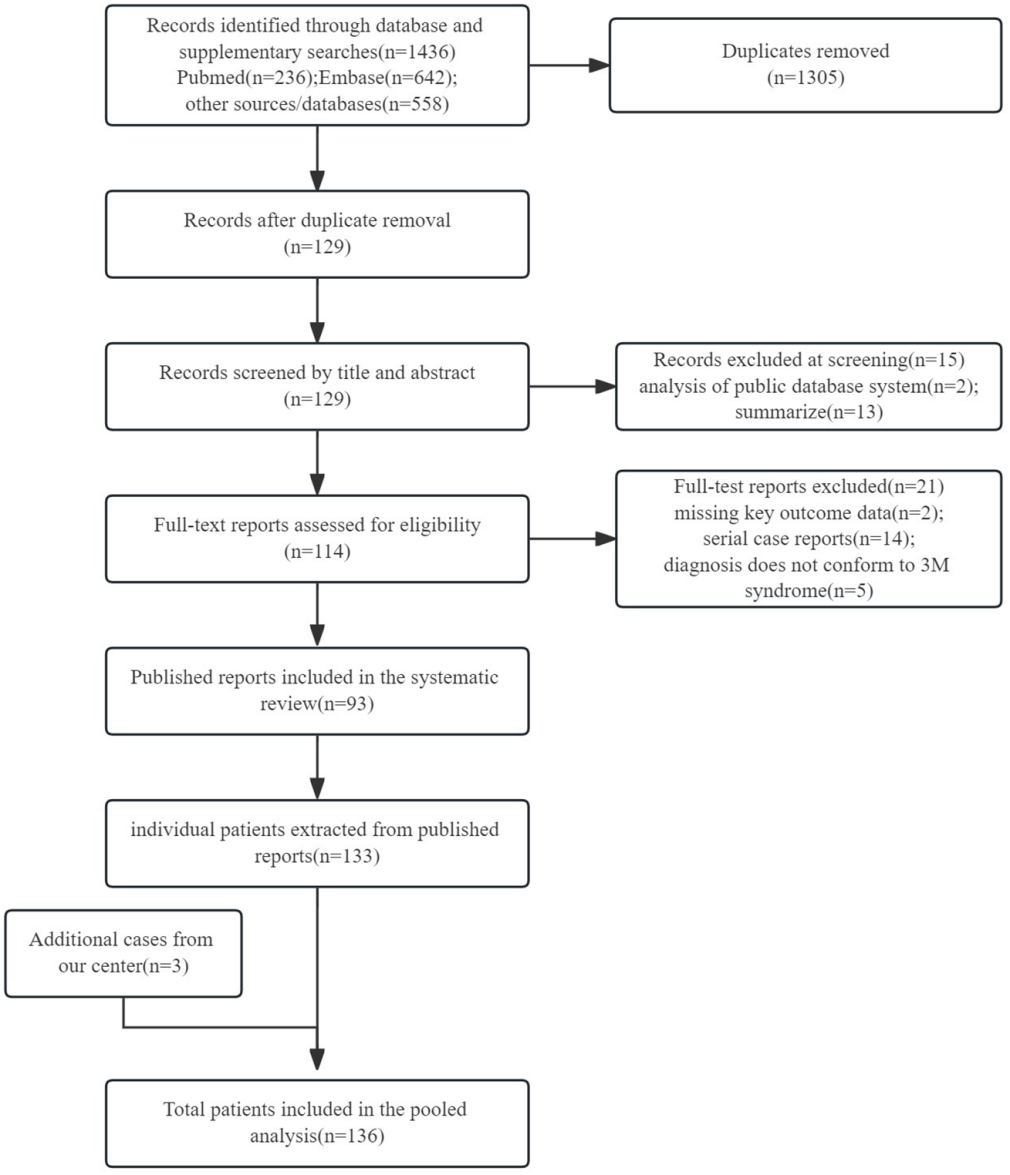
Study selection flow diagram for the systematic review and pooled individual case-level analysis.

Through database and supplementary searches, 1436 records were identified (236 from PubMed, 642 from Embase, and 558 from other databases and supplementary sources). After removal of duplicates, 131 records underwent title and abstract screening, and 106 reports were assessed in full text. Ninety-three published reports were ultimately included, yielding 133 unique patients with ICI-related 3M overlap syndrome after two cases were identified as duplicate publications during revision and removed. Together with three patients from our institution, 136 patients were included in the pooled analysis (**Figure 1**).

### Baseline characteristics

A total of 136 patients were included. Age ranged from 33 to 89 years, and the mean age was 69.8 ± 11.2 years. Among 136 patients with available sex data, 96/136 (70.6%) were male. Melanoma was the most common underlying cancer (40/136, 29.4%), followed by urologic malignancies (35/136, 25.7%) and lung cancer (24/136, 17.6%). Pembrolizumab was the most frequently reported agent (53/136, 39.0%), followed by dual ICI regimens (22/136, 16.2%) and durvalumab (14/136, 10.3%). Dual ICI regimens were defined as concurrent administration of two immune checkpoint inhibitors and were not counted as concomitant non-ICI anticancer therapy. The median time from first ICI exposure to symptom onset was 22.0 days (IQR, 16.0–31.0), and the mean number of treatment cycles before onset was 1.5 ± 0.8.

### Clinical manifestations and ancillary findings

Common manifestations included ptosis (93/136, 68.4%), diplopia (70/136, 51.5%), limb muscle weakness (67/136, 49.3%), dyspnea (60/136, 44.1%), dysphagia (47/136, 34.6%), fatigue (43/136, 31.6%), myalgia (44/136, 32.4%), dysarthria (31/136, 22.8%), and neck muscle weakness (22/136, 16.2%). Most patients presented with multiple symptoms, and 19/136 (14.0%) had the symptom combination of dyspnea plus fatigue. Additional low-frequency symptoms were heterogeneous and were summarized descriptively only.

Ancillary diagnostic findings supporting each component of the 3M overlap syndrome are summarized below; denominators reflect the number of patients with relevant information available, and unreported investigations were not assumed to be negative.

Myocarditis evidence. ECG-related information was available for 109 patients, among whom severe ECG abnormalities (ventricular tachyarrhythmia and/or high-grade atrioventricular block) were reported in 47/109 (43.1%), with complete conduction block documented in 33/109 (30.3%). Reduced left ventricular ejection fraction or systolic dysfunction was reported in 27/77 patients (35.1%) with available echocardiographic data. Cardiac MRI was supportive of myocarditis in 23 patients, and endomyocardial biopsy was supportive in 14/19 patients (73.7%); one additional case was confirmed at autopsy.

Myositis evidence. Creatine kinase elevation was a near-universal finding (median, 28.5-fold above the upper limit of normal). EMG/NCS findings were available for 56 patients, of whom 36 (64.3%) showed myopathic/myositis-type changes; muscle biopsy was supportive in 19/36 patients (52.8%) with reported biopsy results.

MG/NMJ evidence. Acetylcholine receptor antibodies were positive in 39/96 patients (40.6%) with reported testing. Repetitive nerve stimulation and edrophonium/ice-pack testing were performed when available and contributed supportive findings in a subset of cases; results were heterogeneous and are summarized at the patient level in **Supplementary Table 1**.

### Treatment and in-hospital outcomes

Most patients discontinued ICIs and received corticosteroid therapy. Systemic corticosteroids were administered in 133/136 patients (97.8%), and pulse-dose corticosteroids were used in 80/130 (61.5%) of those with available regimen details. Second-line immunomodulatory therapy was given to 119/136 patients (87.5%), comprising IVIG in 91/136 (66.9%), plasma exchange in 41/136 (30.1%), and additional immunosuppressive or targeted immunomodulatory agents in 63/136 (46.3%). Because the timing of second-line therapy was inconsistently reported, upfront versus stepwise escalation could not be reliably distinguished, and treatment escalation patterns are presented descriptively only. Mechanical ventilation was required in 55/131 patients (42.0%) with available data, and pacemaker support was implemented in a subset of patients with high-grade conduction disturbances. No patient met the pre-specified definition of true mechanical circulatory support; vasoactive or inotropic therapy and antiarrhythmic medications were inconsistently reported and could not be pooled quantitatively.

In-hospital outcome was ascertainable for all 136 patients: 53 (39.0%) died during hospitalization and 83 (61.0%) survived to discharge or transfer. Among non-survivors, the median time from symptom onset to in-hospital death was 17.0 days (IQR, 10.0–37.2). Patient-level disposition was heterogeneous and incompletely reported across publications and is summarized descriptively in **Supplementary Table 1**. A summary of the major clinical characteristics and outcomes is presented in **Table 2**.

**Table 2:** Summary of major clinical characteristics and outcomes of patients with ICI-related 3M overlap syndrome.

Characteristic	Value	Characteristic		Value
Age, years	69.8 ± 11.2	Male sex		96/136 (70.6%)
Underlying malignancy				
Melanoma	40/136 (29.4%)	Urologic malignancies		35/136 (25.7%)
Lung cancer	24/136 (17.6%)	Esophageal cancer		10/136 (7.4%)
Thymoma	7/136 (5.1%)	Other		20/136 (14.7%)
ICI agent				
Pembrolizumab	53/136 (39.0%)	Nivolumab		15/136 (11.0%)
Dual ICI regimens	22/136 (16.2%)	Other agents		32/136 (23.5%)
Time from first ICI dose to symptom onset, days	22.0 (16.0–31.0)	Treatment cycles before onset		1.5 ± 0.8
Comorbidities (among 79 patients with available data; full-cohort denominator in brackets)				
Hypertension	43/79 (54.4%) [43/136, 31.6%]	Diabetes mellitus		20/79 (25.3%) [20/136, 14.7%]
Chronic kidney disease	9/79 (11.4%) [9/136, 6.6%]	Coronary artery disease/ischemic heart disease		9/79 (11.4%) [9/136, 6.6%]
Obesity	3/79 (3.8%) [3/136, 2.2%]	At least one reported comorbidity		66/79 (83.5%)
Concomitant non-ICI anticancer therapy (among 59 patients with available data)				
ICI alone	32/59 (54.2%)		ICI + chemotherapy	12/59 (20.3%)
ICI + chemoradiotherapy	3/59 (5.1%)		ICI + targeted/anti-VEGF/TKI	12/59 (20.3%)
Common symptoms				
Ptosis	93/136 (68.4%)	Diplopia		70/136(51.5%)
Limb muscle weakness	67/136 (49.3%)	Dyspnea		60/136 (44.1%)
Dysphagia	47/136 (34.6%)	Fatigue		43/136 (31.6%)
Myalgia	44/136 (32.4%)	Dysarthria		31/136 (22.8%)
Neck muscle weakness	22/136 (16.2%)	Chest tightness		11/136 (8.1%)
Palpitations	10/136 (7.4%)	Dyspnea + fatigue		19/136 (14.0%)
Ancillary findings				
AChR antibody positivity	39/96 (40.6%)	Severe ECG abnormality		47/109 (43.1%)
Reduced LVEF or systolic dysfunction	27/77 (35.1%)	Complete conduction block		33/109 (30.3%)
Treatment and outcomes				
Systemic corticosteroids	133/136 (97.8%)	Pulse-dose corticosteroids		80/130 (61.5%)
IVIG	91/136 (66.9%)	Plasma exchange		41/136 (30.1%)
Other immunosuppressive/targeted agents	63/136 (46.3%)	Mechanical ventilation		55/131 (42.0%)
In-hospital mortality	53/136 (39.0%)			

AChR, acetylcholine receptor; ECG, electrocardiogram; ICI, immune checkpoint inhibitor; IQR, interquartile range; IVIG, intravenous immunoglobulin; LVEF, left ventricular ejection fraction; PLEX, plasma exchange; SD, standard deviation; TKI, tyrosine kinase inhibitor; VEGF, vascular endothelial growth factor.

### Factors associated with in-hospital mortality

Among the 136 patients with available hospitalization outcome data, 53 died in hospital and 83 survived to discharge. In univariable logistic regression, older age (OR, 1.09 per year; 95%CI, 1.04-1.13; P < 0.001), fatigue (OR, 2.41; 95%CI, 1.15-5.06; P = 0.020), and the symptom combination of dyspnea plus fatigue (OR, 2.46;95% CI, 0.92–6.58; P = 0.074), complete conduction block (OR 2.46; 95% CI, 1.07–5.68; P = 0.034), receipt of second-line immunomodulatory therapy (OR 0.30; 95% CI, 0.10–0.86; P = 0.025), and mechanical ventilation (OR 2.39; 95% CI, 1.16–4.91; P = 0.018) were associated with in-hospital mortality. Tumor type, ICI class, the number of ICI doses before onset, the time from first ICI exposure to symptom onset, the time from symptom onset to irAE-directed treatment, and the major comorbidity categories were not significantly associated with in-hospital mortality. In the pre-specified multivariable logistic regression model, which included age, fatigue, and complete conduction block (**Table 1**), older age (adjusted OR [aOR], 1.11 per year; 95% CI, 1.05–1.17; P < 0.001) and complete conduction block (aOR, 2.84; 95% CI, 1.10–7.34; P = 0.031) remained independently associated with in-hospital mortality, whereas fatigue was no longer statistically significant after adjustment (aOR, 1.85; 95% CI, 0.75–4.56; P = 0.181). Mechanical ventilation and receipt of second-line immunomodulatory therapy were not entered into the main multivariable model for the reasons specified in the Methods. All univariable and multivariable analyses are interpreted as exploratory and hypothesis-generating.

### Symptom correlations and hierarchical clustering

**Figure 2:**
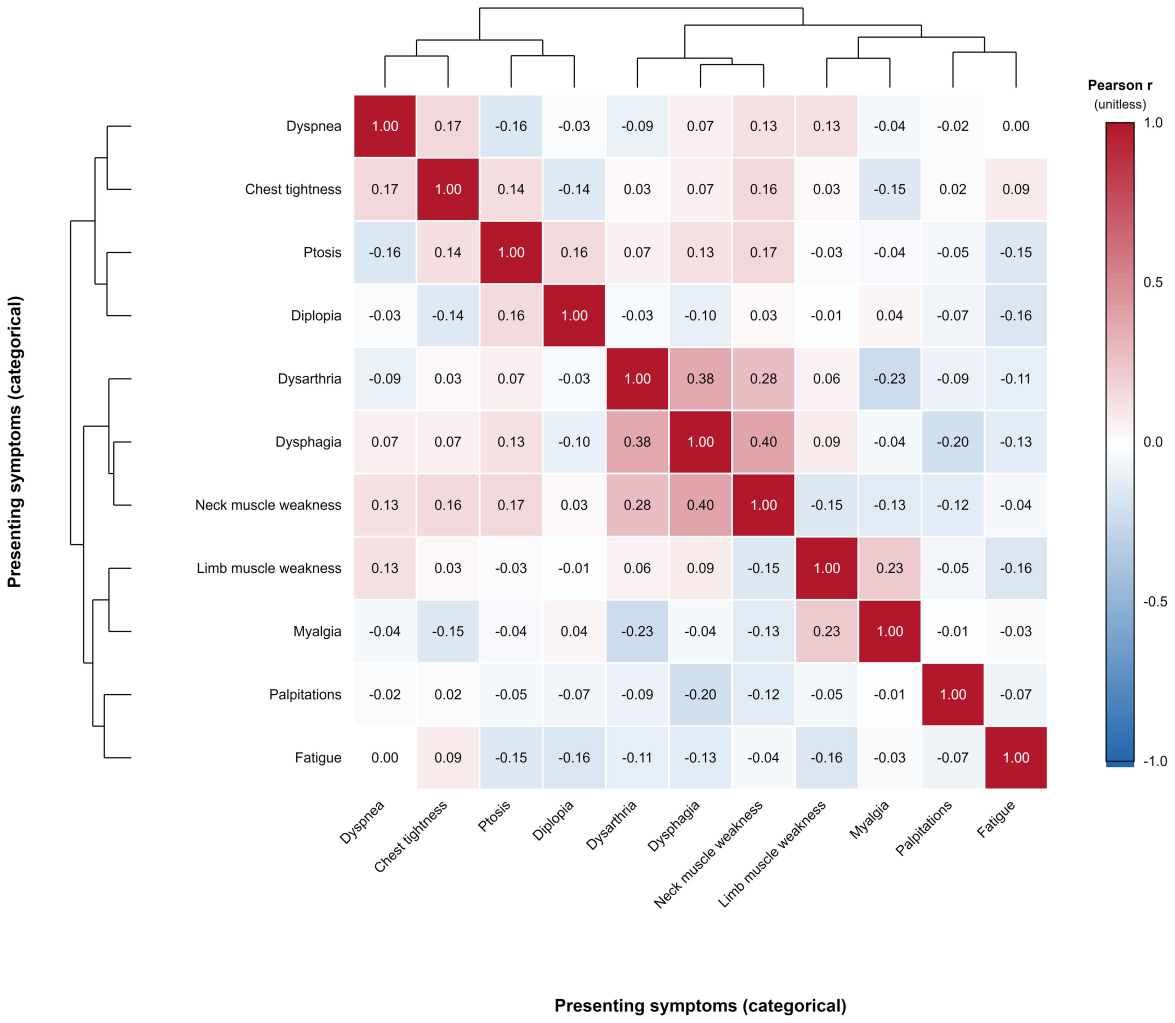
Heatmap of pairwise symptom correlations with hierarchical clustering in patients with ICI-related 3M overlap syndrome.

Exploratory analyses of symptom correlations showed that most symptom pairs were only weakly correlated, underscoring the marked clinical heterogeneity of ICI-related 3M overlap syndrome. Nevertheless, several bulbar and axial muscle symptoms showed moderate and statistically significant co-occurrence. Dysphagia was correlated with neck muscle weakness (r=0.40,P<0.001) and with dysarthria(r=0.38,P<0.001), and dysarthria also correlated with neck muscle weakness (r=0.29,P=0.001). Limb muscle weakness showed a weak but significant correlation with myalgia (r= 0.23,P = 0.007). Other symptom pairs, including those involving fatigue and palpitations, did not reach statistical significance. Hierarchical clustering further suggested that dysphagia, dysarthria, and neck muscle weakness tended to cluster together, consistent with a bulbar/axial-predominant symptom cluster, whereas limb muscle weakness and myalgia formed a weaker cluster suggestive of a more skeletal muscle–predominant phenotype. In contrast, dyspnea, palpitations, and chest discomfort did not cluster tightly with the neuromuscular symptom group. Overall, these findings support substantial clinical heterogeneity, while suggesting that certain symptom co-occurrence patterns may still be clinically informative. The clustering heatmap is shown in **Figure 2**.

## Discussion

In this systematic review of case reports and case series, together with three institutional cases, we characterized 136 patients with ICI-related 3M overlap syndrome and explored factors associated with in-hospital mortality using pooled individual case data. Compared with previous descriptive reviews, the main added value of this study is the exploratory assessment of mortality-associated factors and symptom co-occurrence patterns at the individual case level. Several findings merit emphasis. First, the syndrome was typically an early-onset and clinically fulminant irAE, with a high in-hospital mortality rate of 39%. Second, older age and complete atrioventricular block, recorded as complete conduction block in the extracted dataset, were associated with poorer in-hospital outcomes after adjustment. Fatigue was associated with mortality in univariable analysis but not after adjustment. Finally, exploratory symptom correlation and hierarchical clustering analyses suggested a recurrent bulbar/axial symptom constellation involving dysphagia, dysarthria, and neck muscle weakness. Given the case-level structure of the source data, variable completeness of reporting, and the limited number of outcome events, all mortality-associated findings should be interpreted as exploratory and hypothesis-generating rather than as predictors.

Our epidemiologic findings were broadly consistent with earlier reviews (8, 9). Patients were predominantly older men, melanoma was the most frequently reported malignancy, and symptom onset usually occurred early after ICI initiation, often within the first 1–2 treatment cycles, with a median interval of 22 days from the first ICI exposure to symptom onset. The early onset and fulminant course observed in our cohort are also consistent with prior reports of ICI-related myocarditis and cardiotoxicity (10, 11), supporting the need for heightened vigilance soon after ICI exposure (12, 13). Clinically, new-onset fatigue, dyspnea, ocular symptoms, dysphagia, dysarthria, neck weakness, limb weakness, myalgia, palpitations, or chest discomfort emerging early during ICI therapy should prompt evaluation for possible overlap toxicity rather than being attributed solely to infection, tumor progression, paraneoplastic symptoms, or general treatment burden. Suspicion of one component of the triad should also prompt active assessment for the other two components, including cardiac, skeletal muscle, neuromuscular junction, and respiratory evaluation when clinically indicated. However, the epidemiologic distributions in this pooled dataset should be interpreted cautiously. The predominance of melanoma and of PD-1/PD-L1–directed therapy may partly reflect historical prescribing patterns and broader clinical use of these agents rather than true disease-specific susceptibility. Accordingly, our data are better suited to describing the reported clinical spectrum of the syndrome than to inferring comparative risk across tumor types or drug classes.

Older age was the most robust factor associated with in-hospital mortality. The association remained significant in both univariable and multivariable models, with an approximately 9%–11% increase in the odds of in-hospital death per additional year of age. Although dedicated prognostic studies of 3M overlap syndrome remain scarce, this finding is concordant with earlier reports showing that overlap cases disproportionately affect older adults and that poorer outcomes in ICI-related myocarditis are more common in older patients (9, 14–16). From a clinical perspective, older patients may have less cardiopulmonary reserve, reduced tolerance of respiratory muscle weakness, and lower overall physiologic reserve when myocardial injury, skeletal muscle involvement, and neuromuscular junction dysfunction occur simultaneously. Common cardiometabolic comorbidities, including hypertension, diabetes mellitus, chronic kidney disease, coronary artery disease/ischemic heart disease, and obesity, are highly prevalent in patients receiving ICIs and may further modulate baseline cardiovascular and neuromuscular reserve. In our cohort, hypertension, diabetes mellitus, and ischemic heart disease were the most commonly documented conditions among patients with available comorbidity information, although a substantial proportion of source publications did not explicitly report comorbidity status; unreported comorbidities should not be interpreted as absent. Our findings therefore support a lower threshold for continuous telemetry, serial cardiac biomarker assessment, and close respiratory monitoring in older patients with suspected 3M overlap syndrome.

Complete atrioventricular block was independently associated with in-hospital mortality after adjustment. This finding is clinically plausible (17) and reinforces ECG as a low-cost, widely available bedside tool for early risk stratification. In the context of 3M overlap syndrome, complete atrioventricular block may reflect extensive myocardial and conduction-system inflammation, electrical instability, and a fulminant disease course. The development of new complete atrioventricular block in a patient with suspected 3M overlap syndrome should trigger urgent escalation, including continuous telemetry, early cardiology involvement, consideration of temporary pacing when clinically indicated, and prompt initiation or escalation of immunomodulatory therapy (18, 19).

Fatigue was associated with poorer in-hospital outcomes in univariable analysis, but this association did not remain statistically significant after adjustment for age and complete conduction block. Rather than dismissing fatigue as a nonspecific constitutional complaint, clinicians should recognize it as a potential composite signal reflecting systemic inflammation, skeletal muscle involvement, respiratory muscle weakness, cardiac dysfunction, or diminished physiologic reserve. When fatigue is accompanied by dyspnea, broader organ involvement and more advanced functional compromise may already be present. In the setting of recent ICI exposure, this symptom combination should prompt simultaneous evaluation for cardiac involvement, skeletal muscle injury, neuromuscular junction dysfunction, pulmonary toxicity, infection, thromboembolism, and tumor progression. Mechanical ventilation was excluded from the main multivariable model because it likely lies on the causal pathway between severe 3M overlap syndrome and death, and adjusting for it would risk overcontrol bias.

Our exploratory symptom correlation and hierarchical clustering analyses further highlighted the heterogeneity of this syndrome. Most symptom pairs were only weakly correlated, but several bulbar and axial symptoms showed moderate, statistically significant co-occurrence: dysphagia clustered with neck muscle weakness and dysarthria, suggesting that a bulbar/axial-predominant phenotype may recur in a subset of patients, while limb muscle weakness clustered weakly with myalgia, suggesting a more skeletal muscle–predominant phenotype. These observations are hypothesis-generating rather than definitive, but they may aid clinical pattern recognition in early evaluation. The available pathological data complement these clinical findings. Skeletal muscle biopsy commonly showed immune-mediated necrotizing myositis or myopathy-like changes, whereas myocardial biopsy more often demonstrated active lymphocytic myocarditis. Together, these findings reinforce that the 3M overlap syndrome represents a fulminant immune-inflammatory process with substantive tissue injury rather than a mere coexistence of laboratory abnormalities (20–22).

The apparent association between second-line immunomodulatory therapy and lower in-hospital mortality in univariable analysis should be interpreted with particular caution. This exposure was not included in the primary multivariable model because it cannot be interpreted as a causal treatment effect in retrospective case-level data. Second-line therapy is time-dependent, because patients must survive long enough to receive IVIG, PLEX, or additional immunosuppressive agents, whereas those with the most fulminant courses may die before escalation is possible. In addition, treatment allocation is strongly influenced by confounding by indication, and the timing, sequence, dose intensity, and escalation thresholds of second-line therapy were inconsistently reported. Therefore, the current dataset was insufficient to demonstrate robust treatment–outcome differences, and the univariable association should be viewed as a hypothesis-generating signal supporting prospective evaluation of early combination immunomodulation rather than as evidence that such treatment improves survival. In practice, early ICI interruption, prompt corticosteroid therapy, close cardiac and respiratory monitoring, and timely escalation to IVIG, plasma exchange, or other immunomodulatory strategies remain central to management, particularly in patients with concurrent myocarditis, MG features, respiratory compromise, or complete atrioventricular block (23, 24).

This study has several limitations. First, all pooled data were derived from published case reports and case series, which are inherently vulnerable to publication bias and may overrepresent severe or atypical presentations. Second, the retrospective nature of the source reports resulted in missing or inconsistently reported data, particularly for comorbidities, concomitant non-ICI anticancer therapy, ECG/EMG/MRI/biopsy findings, treatment timing, length of stay, and disposition; unreported items were not assumed to be negative or absent, and denominators therefore vary across variables. Third, although we systematically rechecked all included reports during revision and removed two duplicate publications, residual overlap between publications cannot be fully excluded. Fourth, given the case-level data structure, the limited number of outcome events, and multiple comparisons, all univariable and multivariable analyses are exploratory and hypothesis-generating, and the multivariable model was deliberately constrained to a small number of variables. Fifth, the exploratory clustering analysis was based on binary symptom recording and is susceptible to missingness and reporting bias. Sixth, low-frequency presenting symptoms were heterogeneous and were summarized descriptively rather than entered into mortality models. Larger prospective registries and multicenter cohorts with standardized data collection are needed to validate the factors associated with in-hospital mortality identified here.

## Conclusions

In conclusion, ICI-related 3M overlap syndrome is an early-onset, fulminant irAE with substantial in-hospital mortality. Older age and complete conduction block were independently associated with poorer in-hospital outcomes, whereas fatigue, including the symptom combination of dyspnea plus fatigue, was associated with mortality in univariable but not in multivariable analysis. These findings should be interpreted as exploratory mortality-associated factors rather than predictors, and require validation in prospective registries or multicenter cohorts. Exploratory clustering also suggests that certain bulbar and axial symptoms may co-occur in a nonrandom manner despite the overall heterogeneity of the syndrome. Early recognition of high-risk symptom patterns and rapid multidisciplinary management may be crucial to improving outcomes.

## Data Availability

The raw data supporting the conclusions of this article will be made available by the authors, without undue reservation.
