# Supplementary material for: Immune checkpoint inhibitor–related myositis, myocarditis, and myasthenia gravis overlap syndrome: a systematic review and pooled analysis of individual cases
Source: Front Immunol. 2026 Jul 29;17:1854723. doi: 10.3389/fimmu.2026.1854723 (PMC13461781; doi:10.3389/fimmu.2026.1854723)
Supplement: Supplementary file 1 [file Supplementaryfile1.docx]

**Supplementary Appendix 1. Detailed search strategies for electronic databases**

A comprehensive literature search was conducted in PubMed and Embase from January 1, 2010 to August 31, 2025. Search strategies combined controlled vocabulary terms and free-text terms related to immune checkpoint inhibitors, myocarditis, myositis, myasthenia gravis, overlap syndrome, and case-based publications. Considering the rarity of immune checkpoint inhibitor-related myocarditis, myositis, and myasthenia gravis overlap syndrome and the inconsistency in terminology across reports, a sensitive search strategy was adopted to maximize case capture. The final eligibility of retrieved records was determined by manual screening.

## 1. PubMed

- Date of search: [insert date]
- Time limit: January 1, 2010 to August 31, 2025
- Filters: Humans
- #1 "Immune Checkpoint Inhibitors"[Mesh]
- #2 "Programmed Cell Death 1 Receptor"[Mesh]
- #3 "Programmed Cell Death 1 Ligand 1 Protein"[Mesh]
- #4 "CTLA-4 Antigen"[Mesh]
- #5 checkpoint inhibitor*[tiab]
- #6 immune checkpoint inhibitor*[tiab]
- #7 PD-1 inhibitor*[tiab]
- #8 PD-1 blockade[tiab]
- #9 PD-L1 inhibitor*[tiab]
- #10 PD-L1 blockade[tiab]
- #11 CTLA-4 inhibitor*[tiab]
- #12 CTLA-4 blockade[tiab]
- #13 nivolumab[tiab]
- #14 pembrolizumab[tiab]
- #15 cemiplimab[tiab]
- #16 dostarlimab[tiab]
- #17 toripalimab[tiab]
- #18 sintilimab[tiab]
- #19 camrelizumab[tiab]
- #20 tislelizumab[tiab]
- #21 serplulimab[tiab]
- #22 spartalizumab[tiab]
- #23 retifanlimab[tiab]
- #24 atezolizumab[tiab]
- #25 durvalumab[tiab]
- #26 avelumab[tiab]
- #27 envafolimab[tiab]
- #28 ipilimumab[tiab]
- #29 tremelimumab[tiab]
- #30 #1 OR #2 OR #3 OR #4 OR #5 OR #6 OR #7 OR #8 OR #9 OR #10 OR #11 OR #12 OR #13 OR #14 OR #15 OR #16 OR #17 OR #18 OR #19 OR #20 OR #21 OR #22 OR #23 OR #24 OR #25 OR #26 OR #27 OR #28 OR #29
- #31 "Myocarditis"[Mesh]
- #32 myocarditis[tiab]
- #33 immune-related myocarditis[tiab]
- #34 ICI myocarditis[tiab]
- #35 #31 OR #32 OR #33 OR #34
- #36 "Myositis"[Mesh]
- #37 myositis[tiab]
- #38 myopathy[tiab]
- #39 inflammatory myopathy[tiab]
- #40 immune-related myositis[tiab]
- #41 ICI myositis[tiab]
- #42 #36 OR #37 OR #38 OR #39 OR #40 OR #41
- #43 "Myasthenia Gravis"[Mesh]
- #44 myasthenia gravis[tiab]
- #45 myasthenic[tiab]
- #46 myasthenic crisis[tiab]
- #47 immune-related myasthenia gravis[tiab]
- #48 ICI myasthenia gravis[tiab]
- #49 #43 OR #44 OR #45 OR #46 OR #47 OR #48
- #50 overlap syndrome[tiab]
- #51 overlap syndrom*[tiab]
- #52 3M syndrome[tiab]
- #53 IM3OS[tiab]
- #54 myo-carditis[tiab]
- #55 #50 OR #51 OR #52 OR #53 OR #5456
- #56 "Case Reports"[Publication Type]
- #57 case report[tiab]
- #58 case reports[tiab]
- #59 case series[tiab]
- #60 case-series[tiab]
- #61 case based[tiab]
- #62 #56 OR #57 OR #58 OR #59 OR #60 OR #61
- #63 (#35 AND #42 AND #49)
- #64 (#35 AND #42)
- #65 (#35 AND #49)
- #66 (#42 AND #49)
- #67 (#63 OR #64 OR #65 OR #66 OR #55)
- #68 #30 AND #67 AND #62
- 236篇

## 2. Embase

- Database platform: Embase (Elsevier)
- Date of search: [insert date]
- Time limit: January 1, 2010 to August 31, 2025
- Limits: Human
- #1 'immune checkpoint inhibitor'/exp
- #2 'programmed cell death 1 receptor'/exp
- #3 'programmed death ligand 1'/exp
- #4 'cytotoxic t lymphocyte associated antigen 4'/exp
- #5 'nivolumab'/exp
- #6 'pembrolizumab'/exp
- #7 'cemiplimab'/exp
- #8 'dostarlimab'/exp
- #9 'toripalimab'/exp
- #10 'sintilimab'/exp
- #11 'camrelizumab'/exp
- #12 'tislelizumab'/exp
- #13 'serplulimab'/exp
- #14 'atezolizumab'/exp
- #15 'durvalumab'/exp
- #16 'avelumab'/exp
- #17 'ipilimumab'/exp
- #18 'tremelimumab'/exp
- #19 checkpoint inhibitor*:ti,ab,kw
- #20 immune checkpoint inhibitor*:ti,ab,kw
- #21 PD-1 inhibitor*:ti,ab,kw
- #22 PD-L1 inhibitor*:ti,ab,kw
- #23 CTLA-4 inhibitor*:ti,ab,kw
- #24 #1 OR #2 OR #3 OR #4 OR #5 OR #6 OR #7 OR #8 OR #9 OR #10 OR #11 OR #12 OR #13 OR #14 OR #15 OR #16 OR #17 OR #18 OR #19 OR #20 OR #21 OR #22 OR #23
- #25 'myocarditis'/exp
- #26 myocarditis:ti,ab,kw
- #27 'immune-related myocarditis':ti,ab,kw
- #28 #25 OR #26 OR #27
- #29 'myositis'/exp
- #30 myositis:ti,ab,kw
- #31 myopathy:ti,ab,kw
- #32 'inflammatory myopathy':ti,ab,kw
- #33 'immune-related myositis':ti,ab,kw
- #34 #29 OR #30 OR #31 OR #32 OR #33
- #35 'myasthenia gravis'/exp
- #36 'myasthenia gravis':ti,ab,kw
- #37 myasthenic:ti,ab,kw
- #38 'myasthenic crisis':ti,ab,kw
- #39 'immune-related myasthenia gravis':ti,ab,kw
- #40 #35 OR #36 OR #37 OR #38 OR #39
- #41 'overlap syndrome':ti,ab,kw
- #42 '3M syndrome':ti,ab,kw
- #43 IM3OS:ti,ab,kw
- #44 myo-carditis:ti,ab,kw
- #45 #41 OR #42 OR #43 OR #44
- #46 'case report'/exp
- #47 'case report':ti,ab,kw
- #48 'case reports':ti,ab,kw
- #49 'case series':ti,ab,kw
- #50 'case-series':ti,ab,kw
- #51 #46 OR #47 OR #48 OR #49 OR #50
- #52 (#28 AND #34 AND #40)
- #53 (#28 AND #34)
- #54 (#28 AND #40)
- #55 (#34 AND #40)
- #56 #52 OR #53 OR #54 OR #55 OR #45
- #57 #24 AND #56 AND #51
- 642篇
- Cochrone 14条
- 万方272条

## Notes

- Search strategies were adapted to the indexing system and syntax of each database.
- Additional manual screening of the reference lists of included studies was performed to identify potentially eligible reports not captured by electronic database searches.
- Google Scholar was used as a supplementary source for citation tracking and additional retrieval.
- The full eligibility assessment was based on predefined inclusion and exclusion criteria and performed by manual review of titles, abstracts, and full texts.
