# Supplementary material for: Immune checkpoint inhibitor–related myositis, myocarditis, and myasthenia gravis overlap syndrome: a systematic review and pooled analysis of individual cases
Source: Front Immunol. 2026 Jul 29;17:1854723. doi: 10.3389/fimmu.2026.1854723 (PMC13461781; doi:10.3389/fimmu.2026.1854723)
Supplement: Supplementary file 2 [file Table1.docx]

**Supplementary Table 1. Patient-level characteristics, diagnostic evidence, treatment, and outcomes of published cases of ICI-related myocarditis–myositis–myasthenia gravis overlap syndrome.**

| **Case ID** | **First author, year** | **Country/region** | **Age** | **Sex** | **Cancer type** | **ICI regimen** | **Concomitant non-ICI anticancer therapy** | **Time from ICI initiation to symptom onset** | **Time from presentation to irAE-directed treatment** | **Reported comorbidities** | **Myocarditis evidence** | **Myositis evidence** | **MG/NMJ evidence** | **Treatment summary** | **Supportive/cardiac interventions** | **Outcome/disposition** |
| --- | --- | --- | --- | --- | --- | --- | --- | --- | --- | --- | --- | --- | --- | --- | --- | --- |
| P001 | Jurii Karibe, 2025 | Japan | 76 | Female | Pancreatic metastasis of renal cell carcinoma | Avelumab | Axitinib | 28 | NR | Hypertension; dyslipidemia | Chest pain/dizziness; Tn↑; CMR− | Weakness/fatigue; CK↑; EMG+ | Ptosis/dysphagia; AChR+ | Pulse steroids | None reported | Improved; discharged home |
| P002 | Hiroki Kamikawa, 2025 | Japan | 80 | Male | Muscle-invasive bladder cancer | Nivolumab | None | 18 | 1 day | Hypertension; thyroid cancer | Tn↑; ECG: complete AV block/VT; CAG−; EMB+ | Proximal weakness; CK↑ | Ptosis/diplopia/ophthalmoplegia; AChR+ | Pulse steroids; IVIG; PLEX; TAC | Pacemaker | Death due to multiple organ failure |
| P003 | Mette Syberg Jespersen, 2021 | Denmark | 57 | Male | Renal cell carcinoma | Nivolumab plus ipilimumab | None | 12 | NR | NR | Tn↑; ECG: AV block/RBBB/VT; Echo− | Myalgia/weakness; CK↑; EMG+ | Diplopia/ptosis; AChR+ | Steroids; ABA; MMF | Pacemaker | Improved; discharged |
| P004 | Matthew T. Newman, 2024 | USA | 87 | Male | Advanced urothelial carcinoma | Pembrolizumab | None | 21 | NR | NR | Tn↑; ECG−; Echo− | Fatigue/body pain; CK↑ | Ptosis/ophthalmoplegia; AChR− | Steroids; PYR; IVIG; RTX | None reported | Death after no further escalation of care |
| P005 | Shoichiro Saito, 2024 | Japan | 78 | Male | Non-small-cell lung carcinoma | Durvalumab | Radiotherapy; chemotherapy | 32 | 4 days | NR | Dyspnea; Tn↑; ECG: ST changes; Echo+; CAG− | Pain/weakness; CK↑; EMG+ | Respiratory involvement; AChR+; anti-titin+; RNS+ | PLEX | None reported | Improved; discharged |
| P006 | Jing Bai, 2022 | China | 69 | Male | Esophageal squamous cell carcinoma | Camrelizumab | Chemotherapy | 21 | NR | Diabetes mellitus | Palpitations; Tn↑; ECG: sinus tachycardia | Lower-limb weakness/myalgia; CK↑; EMG+ | Ptosis/ocular movement limitation; AChR+ | Steroids; PYR; IVIG | None reported | Improved; discharged |
| P007 | Yue-Bei Luo, 2021 | China | 47 | Female | Bone metastasis of thymoma; no prior MG | Toripalimab | None | 28 | NR | NR | Dyspnea; Tn↑; ECG: RBBB/third-degree AV block | Myalgia/weakness; CK↑; EMG+ | Diplopia/dysphagia; AChR+; RyR+; RNS− | PYR | Pacemaker | Improved; discharged |
| P008 | Femi Williams Adeoye, 2024 | United Kingdom | 69 | Female | Lung adenocarcinoma | Durvalumab | Chemotherapy plus radiotherapy | 28 | NR | NR | Dyspnea; Tn↑; ECG: bradycardia/third-degree AV block | Weakness/myalgia; CK↑; EMG+; muscle MRI+ | Diplopia; AChR−; RNS− | Steroids; IVIG; colchicine; ramipril | Pacemaker | Improved; discharged |
| P010 | Loulu Gao, 2021 | China | 67 | Female | Rectal neuroendocrine tumor | Camrelizumab | Sorafenib | 31 | NR | Diabetes mellitus | Chest tightness/dyspnea; Tn↑; ECG: arrhythmia/AV conduction abnormality; Echo+ | Weakness; CK↑ | Ptosis | Steroids; IVIG; anticholinesterase therapy; myocardial supportive therapy | NR | Treatment withdrawn; discharged against medical advice; subsequently died |
| P011 | Nadine A. Abdeljabbar, 2024 | USA | 77 | Female | Bladder cancer | Pembrolizumab | None | 14 | NR | NR | Tn↑; ECG: ST changes; CMR+ | Myalgia; CK↑; muscle MRI+ | Ptosis; NMJ details NR | Steroids; PLEX; IVIG | NR | Improved; discharged home |
| P012 | T. Cooksley, 2023 | United Kingdom | 66 | Female | Cholangiocarcinoma | Pembrolizumab | NR | 7 | NR | NR | Fatigue/nausea; Tn↑; ECG−; Echo+; CMR performed | Fatigue; CK↑ | Ptosis; AChR weak+ | Steroids; IVIG; MMF; ABA; PYR | NR | Discharged on day 7 |
| P014 | Chelby Wakefield, 2021 | USA | 55 | Female | Melanoma | Pembrolizumab | None | 21 | NR | NR | Dyspnea; Tn↑; ECG: ST changes/third-degree AV block/VT; CAG−; Echo− | Muscle spasm/weakness | Diplopia/facial droop; AChR+; striational Ab+ | Steroids; PLEX; ABA; IVIG | ICD | Discharged to rehabilitation on day 55 |
| P015 | Ryoko Shibuya, 2024 | Japan | 83 | Male | Hepatocellular carcinoma | Atezolizumab | Bevacizumab | 21 | 1 day | Diabetes mellitus | Dyspnea; Tn↑; ECG: RBBB/ST changes/AF; CAG−; Echo−; EMB+ | Limb weakness; CK↑; EMG+ | Ptosis/dysarthria/dysphagia; AChR+; striational/Kv1.4/titin Ab+ | Pulse steroids; IVIG | None reported | Discharged on day 193 |
| P016 | Monica Mariniello, 2024 | Italy | 40 | Female | Lung adenocarcinoma | Nivolumab plus ipilimumab | Chemotherapy | 30 | Within 48 hours | Hashimoto thyroiditis | Dyspnea; Tn↑; ECG: ST elevation/RBBB; Echo+; CMR+ | Myalgia/neck weakness; CK↑; muscle biopsy+; myositis Ab+ | Diplopia/ophthalmoplegia/ptosis/dysphagia; AChR−; MuSK−; RNS−; ice test+ | Pulse steroids; IVIG; PYR; MTX | NR | Symptoms improved after 3 months; chemotherapy continued |
| P017 | Biji Soman, 2022 | United Kingdom | 70 | Female | Lung adenocarcinoma | Pembrolizumab | None | 21 | Day 4 | Type 2 diabetes mellitus; hypertension; peripheral vascular disease | Dyspnea; Tn↑; ECG: atrial tachycardia/complete heart block; Echo− | Weakness/fatigue; CK↑ | Ptosis/facial droop/blurred vision; AChR+; anti-titin+ | IVIG; anticholinesterase therapy | None reported | Died on day 7 |
| P018 | Beibei Yin, 2022 | China | 71 | Female | Cholangiocarcinoma | Sintilimab | Gemcitabine plus cisplatin | 7 | NR | NR | Mild dyspnea; Tn↑; ECG: atrial rhythm/tachycardia; Echo−; delayed CMR+ | Lower-limb weakness/soreness; CK↑; EMG−; myositis Ab− | Ptosis; AChR weak+; RyR− | Steroids; IVIG; TAC | None reported | Symptoms improved after 3 months; chemotherapy continued |
| P019 | Zeynep Ünlütürk, 2023 | Turkey | 33 | Male | Thymoma | Nivolumab | None | 90 | NR | NR | Cardiac details NR | CK↑; muscle biopsy+; EMG− | Diplopia/ocular movement limitation/slurred speech; AChR− | Steroids; IVIG; AZA; PYR | None reported | Symptoms controlled after 1 month; nivolumab continued with close monitoring |
| P020 | Qian Xing, 2020 | China | 66 | Male | Lung adenocarcinoma and thymoma, type B2 | Sintilimab | None | 25 | NR | NR | Dyspnea; Tn↑; ECG: complete RBBB/conduction block; Echo− | Progressive limb weakness/myalgia; CK↑ | Ptosis/ophthalmoplegia/dysphagia; AChR+ | Pulse steroids; IVIG; PYR; PLEX | Pacemaker | Rehabilitation at 3 months; tumor continued to progress |
| P021 | Berin Inan, 2025 | Turkey | 73 | Male | Cutaneous melanoma | Nivolumab plus ipilimumab | None reported | 25 | NR | NR | Tn↑; ECG−; CAG−; Echo−; CMR− | Dropped head/myalgia/difficulty walking; CK↑; EMG+; muscle biopsy+ | Diplopia/ptosis/fatigue; AChR−; MuSK−; anti-titin+ | Steroids; IVIG; PLEX; RTX; PYR | None reported | Symptoms controlled after 1 month; nivolumab continued with close monitoring |
| P022 | Ryohei Ono, 2022 | Japan | 77 | Female | Lung adenocarcinoma | Nivolumab | None | 22 | 4 days | NR | Tn↑; ECG: AV block/RBBB/ST elevation; CAG−; Echo− | Myalgia; CK↑; EMG−; muscle MRI+ | Ophthalmoplegia/ptosis; AChR−; MuSK−; anti-titin+; Kv1.4+ | Pulse steroids; IVIG | Pacemaker | Died on day 29 due to respiratory failure |
| P023 | Yumi Saishu, 2022 | Japan | 55 | Female | Melanoma with history of thymoma surgery | Nivolumab | None | 28 | Within 24 hours | NR | Tn↑; ECG: PVCs/VT; Echo+ | CK↑; muscle MRI+ | Ocular movement disorder/diplopia/dysphagia; AChR+ | Steroids; IVIG; PLEX | None reported | Discharged to rehabilitation on day 254 |
| P024 | Elena Giovannini, 2023 | Italy | 65 | Male | Melanoma | Pembrolizumab | None reported | 12 | 3 days | NR | Dyspnea/palpitations; Tn↑; ECG: RBBB/AF/VT; CAG+; Echo−; autopsy myocarditis+ | Fever/myalgia; CK↑ | Diplopia/gait instability; PYR used | Steroids; PYR | NR | Died on hospital day 3 due to multiple organ failure |
| P025 | Alfred Schwab, 2022 | USA | 81 | Male | Bladder cancer | Pembrolizumab | None reported | 51 | NR | Hypertension; hypothyroidism | Tn↑; ECG−; Echo− | Limb weakness/pain; CK↑; muscle MRI+; muscle biopsy+ | Dysphagia/diplopia/ptosis; MG serology−; NCS suggests NMJ disorder | Steroids; IVIG; PLEX; RTX; PYR | NR | Continued rehabilitation after 4 weeks |
| P026 | Phillip Taboada, 2024 | USA | 69 | Male | Papillary renal cell carcinoma | Pembrolizumab | Axitinib | 63 | 42 days reported; timing unclear | NR | Bradycardia/conduction disease; ECG: heart block; CAG−; Echo− | Myositis details limited; aldolase↑ | Dysphagia/ptosis/ophthalmoparesis/facial weakness/dysarthria; AChR−; anti-LRP4+ | Pulse steroids; IVIG; PYR | Pacemaker | Discharged with persistent dysphagia and ophthalmoplegia |
| P027 | Arman ÇAKAR, 2023 | Turkey | 63 | Male | Non-small-cell lung cancer | Pembrolizumab | Cisplatin plus pemetrexed | 28 | 5 days | NR | Dyspnea; Tn↑; ECG−; Echo−; CMR− | Generalized/upper-limb weakness; CK↑; EMG+ | Diplopia; MG Ab−; SFEMG+; anti-titin+ | Steroids; IVIG; PYR | None reported | Improved; discharged |
| P028 | Carlota Baptista, 2025 | Portugal | 70 | Female | Mucinous colon adenocarcinoma | Pembrolizumab | None reported | 12 | 4 days | Autoimmune or cardiac disease reported; details unclear | Tn↑; ECG−; CMR+ | Generalized myalgia/weakness; CK↑; EMG+ | Dysphagia/dysarthria; AChR−; PYR used | Steroids; IVIG; PLEX; PYR | None reported | Continued rehabilitation on day 93 |
| P029 | Zi-xuan Yang, 2021 | China | 33 | Male | Thymoma | Sintilimab | None reported | 224 | 10 days | None | Dyspnea/palpitations; Tn↑; ECG: LBBB; Echo: LV dilation with preserved EF | Muscle weakness; CK↑ | Ptosis/dysarthria; PYR used | Steroids; IVIG; PYR | None reported | Improved; discharged |
| P030-1 | John A. Cuenca, 2023 | USA | 62 | Male | Clear cell renal cell carcinoma | Nivolumab plus ipilimumab | None reported | NR | NR | NR | Dyspnea/tachycardia/fatigue; Tn↑; ECG+; CAG+; Echo−; EMB+ | Fatigue; CK↑; EMG+; muscle biopsy+ | NMJ dysfunction not evident on EMG | Pulse steroids; PLEX; RTX | None reported | Symptoms improved after 10 days; discharged home with oral corticosteroid taper |
| P030-2 | John A. Cuenca, 2023 | USA | 76 | Male | Prostate cancer | Nivolumab plus ipilimumab | None reported | 3 days | 3 days | Coronary artery disease | Fever/weakness; Tn↑; ECG+; CAG+; Echo−; EMB+ | Muscle weakness; CK↑; muscle biopsy+ | Dysphagia/dysarthria; neuropathy reported; NMJ details NR | Pulse steroids; PLEX; IFX; RTX | None reported | Discharged after 12 days with oral corticosteroid taper |
| P030-3 | John A. Cuenca, 2023 | USA | 72 | Male | Metastatic squamous cell carcinoma involving inguinal region | Cemiplimab | None reported | 64 | NR | NR | Tn↑; ECG: PVCs; CAG+; Echo−; EMB+ | Lower-limb weakness; CK↑; EMG+ | Dysphagia; MG/paraneoplastic Ab−; PYR used | Pulse steroids; PLEX; IVIG; RTX; PYR | None reported | Continued rehabilitation 1 month after tracheostomy |
| P030-4 | John A. Cuenca, 2023 | USA | 72 | Male | Lung adenocarcinoma | Nivolumab plus ipilimumab | None reported | NR | At diagnosis | COVID-19 | Dyspnea; Tn↑; ECG−; Echo+ | Generalized weakness; CK↑; EMG+ | NMJ details NR | Pulse steroids; PLEX | None reported | Continued rehabilitation after 40 days |
| P031-1 | François Deharo, 2022 | France | 65 | Female | Melanoma | Nivolumab plus relatlimab | None reported | 16 | 6 days | Hypertension; diabetes mellitus; pancreatitis | Dyspnea; Tn↑; ECG: RBBB; Echo−; CMR+ | Fatigue/weakness; EMG−; muscle biopsy+ | Ptosis/diplopia/dysarthria; MG Ab− | Pulse steroids; PLEX; ABA | None reported | Discharged after 50 days; complete symptom resolution at 1 year |
| P031-2 | François Deharo, 2022 | France | 84 | Male | Melanoma | Nivolumab plus ipilimumab | None reported | 19 | 5 days | Hypertension; asthma | Dyspnea; Tn↑; ECG: LBBB/high-grade AV block; Echo− | Myalgia/fatigue; muscle biopsy+ | Ptosis/diplopia/dysphagia | Pulse steroids | None reported | Died after 5 days due to disease progression |
| P031-3 | François Deharo, 2022 | France | 70 | Male | Melanoma | Nivolumab plus ipilimumab and relatlimab | None reported | 35 | 19 days reported; timing unclear | Hypertension; dyslipidemia; diabetes mellitus | Tn↑; ECG: sinus tachycardia/RBBB/high-grade AV block; CAG−; Echo+; CMR+; EMB+ | Proximal weakness; ENMG− | Diplopia/ptosis/dysarthria; AChR−; MuSK− | Pulse steroids; IVIG; IFX; ABA; PLEX | Pacemaker | Died 62 days after the second ICI infusion |
| P032-1 | Alberto Sánchez-Camacho, 2025 | Spain | 75 | Male | Melanoma | Nivolumab | None reported | 15 | NR | NR | Syncope/chest pain/dyspnea; Tn↑; ECG: complete AV block; CAG−; Echo− | Neck weakness; CK↑; EMG+ | Diplopia/ptosis/dysphagia/nasal voice; AChR−; anti-titin weak+; NMJ testing− | Steroids; IVIG; PYR; TCZ; PLEX | Pacemaker | Died 14 days after admission due to multiple organ failure |
| P032-2 | Alberto Sánchez-Camacho, 2025 | Spain | 42 | Female | Melanoma | Pembrolizumab | None | 27 | NR | None | Tn↑; CMR+ | Mild generalized weakness; CK↑; EMG+ | Ptosis; AChR−; anti-titin− | Steroids; IVIG; PYR; TCZ | None reported | Discharged 35 days after first ICI; corticosteroids discontinued after 3 months |
| P032-3 | Alberto Sánchez-Camacho, 2025 | Spain | 59 | Female | Clear cell renal cell carcinoma | Nivolumab | None reported | 35 | NR | Hashimoto thyroiditis | Tn↑; ECG: ST depression/SVPCs; Echo−; CMR+ | Neck weakness; CK↑ | Ptosis/diplopia/dysphagia/dysarthria; AChR−; MuSK−; anti-titin+ | Steroids; IVIG; PYR; PLEX; RTX | None reported | Symptoms improved after 4 months |
| P032-4 | Alberto Sánchez-Camacho, 2025 | Spain | 70 | Male | Gastric adenocarcinoma | Pembrolizumab | None | 21 | NR | NR | Dyspnea; Tn↑; ECG: first-degree AV block; Echo−; CMR+ | Fatigue/myalgia/weakness; CK↑; EMG+ | Ptosis; NMJ testing− | Steroids; IVIG; TCZ | NR | Stable; discharged after 30 days |
| P033-1 | Şule Deveci, 2025 | Turkey | 67 | Male | Small-cell lung cancer | Pembrolizumab | Chemotherapy | 26 | NR | Hypertension; type 2 diabetes mellitus; left bundle branch block | Tn↑; ECG−; Echo− | CK↑; EMG+; orbital MRI+; muscle biopsy+ | Ptosis/ophthalmoplegia; AChR−; MuSK−; RNS reported | Pulse steroids; IVIG; PLEX; RTX; PYR | None reported | Died 2 weeks after second rituximab due to aspiration pneumonia and sepsis |
| P033-2 | Şule Deveci, 2025 | Turkey | 68 | Male | Non-small-cell lung cancer | Pembrolizumab | Chemotherapy | 84 | NR | Hashimoto thyroiditis; ischemic heart disease; hypertension; hyperlipidemia | Tn↑ | Neck weakness; CK↑; EMG+ | Diplopia/ptosis/dysphagia; AChR−; MuSK−; RNS− | IVIG | NR | Pneumonia occurred 1 month later; died 2 months later due to tumor progression |
| P034-1 | Zenia Elavia, 2024 | USA | 71 | Male | Esophageal adenocarcinoma | Nivolumab | Chemotherapy; radiotherapy | 35 | NR | Hypertension; hyperlipidemia; obstructive sleep apnea; acid reflux disease | Tn↑; NSTEMI-like presentation | CK↑; EMG− | Fatigue/weakness/dyspnea; RNS− | Prednisone/steroids; IVIG; PLEX | None reported | Died due to worsening myasthenic symptoms |
| P034-2 | Zenia Elavia, 2024 | USA | 66 | Female | Metastatic squamous cell carcinoma | Cemiplimab | None | 14 | 2 weeks | Hypertension; hyperlipidemia | Myocarditis evidence limited | Weakness in the proximal upper limbs; EMG− | Swallowing difficulties/dysarthria/diplopia/right eye ptosis; AChR−; MuSK−; RNS− | Prednisone; PLEX | NR | Stable; discharged after 10 days |
| P035 | Xiaohong Xie, 2021 | China | 67 | Male | Large-cell lung cancer | Pembrolizumab | Pemetrexed plus carboplatin | 14 | NR | None | Exertional dyspnea; Tn↑; ECG: complete LBBB; CAG−; Echo+ | Limb weakness/paralysis; CK↑; muscle biopsy+ | Ptosis/blurred vision; MG details NR | Steroids | Pacemaker | Required steroids again for immune-related pneumonitis; no recurrence within 3 months |
| P036 | Rafael Valenti-Azcarate, 2019 | Spain | 66 | Male | Small-cell lung cancer | Nivolumab | None | 35 | NR | None | Mild dyspnea; Tn↑; ECG: AF; Echo/CMR: mild LV dysfunction | Fatigue/back pain; CK↑; EMG+; muscle biopsy+ | Diplopia; AChR−; MuSK−; RNS− | Steroids | NR | Improved and discharged; died 2 months later due to tumor progression |
| P037 | Charles Szuchan, 2019 | USA | 70 | Female | Thymoma | Pembrolizumab | None | 16 | NR | NR | Dyspnea; Tn↑; ECG: RBBB/ST changes/complete heart block; Echo+; EMB+ | CK↑ | AChR+; PYR used | Pulse steroids; PLEX; PYR | Pacemaker | Improved and discharged after 50 days |
| P039 | Nikeshan Jeyakumar, 2020 | USA | 86 | Male | Metastatic periorbital squamous cell carcinoma | Cemiplimab | None reported | 21 | 5 days | Hypertension; hyperlipidemia; chronic kidney disease; sick sinus syndrome status post pacemaker; coronary artery disease | Fatigue; Tn↑; ECG: ischemia/RBBB; Echo−; EMB+ | Back/hip pain; CK↑ | Ptosis/ocular involvement; AChR+; striational Ab+ | Pulse steroids; PLEX; IVIG | CRRT | Died due to irAE |
| P040 | Marium Fazal, 2019 | Australia | 82 | Male | Melanoma | Nivolumab | None | 31 | 2 weeks reported; timing unclear | Type 2 diabetes mellitus; hypertension | Tn↑; ECG−; Echo− | Neck stiffness/dropped head/fatigue; CK↑ | Dysarthria/ptosis; PYR used | Pulse steroids; IVIG; PYR | Mechanical ventilation/reintubation | Died after initial extubation followed by reintubation |
| P041-1 | Puja Arora, 2020 | USA | 70 | Male | Metastatic melanoma | Ipilimumab plus nivolumab | None | 11 | NR | Hypertension; history of colon cancer | Palpitations/presyncope; Tn↑; ECG: complete heart block; Echo+; CMR+ | Weakness; CK↑ | Diplopia/ptosis; AChR+ | Steroids; ATG; MMF; cyclophosphamide; PLEX | Pacemaker | Died after 17 days due to heart failure and malignant arrhythmia |
| P041-2 | Puja Arora, 2020 | USA | 79 | Male | Metastatic melanoma | Pembrolizumab | None reported | 26 | NR | Chronic lymphocytic leukemia | Tn↑; ECG: complete heart block; Echo− | Lower-limb weakness/diffuse pain; CK↑ | Blurred vision/diplopia; AChR− | Pulse steroids; ATG; MMF; cyclophosphamide; IVIG | Pacemaker | Died after 21 days due to gastrointestinal bleeding and pulmonary embolism |
| P041-3 | Puja Arora, 2020 | USA | 61 | Female | Metastatic breast cancer | Durvalumab plus tremelimumab | None reported | 28 | NR | NR | Tn↑; ECG: PACs/QTc prolongation; Echo−; CMR− | CK↑ | Ptosis; AChR− | Steroids; MMF; PYR | None reported | Died after 41 days |
| P041-4 | Puja Arora, 2020 | USA | 69 | Male | Metastatic urothelial carcinoma | Pembrolizumab | None | 132 | NR | Chronic kidney disease; hypertension; hyperlipidemia; type 2 diabetes mellitus; coronary artery disease | Tn↑; NSTEMI-like presentation; CAG−; Echo+ | Diffuse pain/weakness; CK↑ | MG/NMJ details NR | Steroids; MMF; ATG | NR | Died after 17 days |
| P041-5 | Puja Arora, 2020 | USA | 67 | Female | Metastatic melanoma | Ipilimumab plus nivolumab | None | 14 | NR | NR | Dyspnea; Tn↑; Echo−; CMR− | Diffuse weakness; CK↑ | Dysphagia; AChR+ | Steroids; MMF; ATG; PLEX | None reported | Died after 34 days |
| P041-6 | Puja Arora, 2020 | USA | 83 | Male | Melanoma | Nivolumab | None reported | 31 | NR | Hypertension; hyperlipidemia; atrial fibrillation | Chest pain/orthopnea; Tn↑; cardiac details limited | Fatigue/weakness; CK↑ | Dysphagia/ptosis/ocular abnormalities; AChR− | Steroids; colchicine/naproxen; PLEX | Mechanical ventilation reported | Died after 42 days due to respiratory failure |
| P041-7 | Puja Arora, 2020 | USA | 70 | Male | Metastatic renal cell carcinoma | Ipilimumab plus nivolumab | None reported | 21 | NR | Hypertension; chronic kidney disease; atrial fibrillation | Tn↑; ECG−; Echo− | Generalized weakness/fatigue; CK↑ | AChR−; MG/NMJ details limited | Pulse steroids; PLEX; IFX | None reported | Died after 67 days due to gastrointestinal bleeding |
| P041-8 | Puja Arora, 2020 | USA | 89 | Male | Non-small-cell lung cancer | Pembrolizumab | None reported | 32 | NR | Hypertension; hyperlipidemia; coronary artery disease; chronic kidney disease; type 2 diabetes mellitus | Tn↑; ECG: NSVT/high-grade AV block; Echo+; CMR− | Dysphagia/imbalance; CK↑; EMG+ | Disconjugate gaze/blurred vision/dysphagia; AChR− | Steroids | NR | Died after 21 days due to refractory myasthenic symptoms |
| P042-1 | Yukai Chen, 2021 | China | 77 | Male | Chordoma | Sintilimab | Anlotinib | 21 | NR | None | Chest tightness/dyspnea; Tn↑; ECG: ST-T changes; Echo+; PET-MRI+ | Lower-limb weakness; CK↑ | Ptosis/ophthalmoplegia; AChR+ | Steroids | None reported | Discharged against medical advice after 15 days; died 3 days later due to infection |
| P042-2 | Yukai Chen, 2021 | China | 69 | Female | Non-small-cell lung cancer | Camrelizumab | Chemotherapy | 20 | NR | Hypertension | Palpitations; Tn↑; ECG: sinus tachycardia/atrial arrhythmia/ST depression; Echo+ | Myositis evidence limited; muscle enzymes↑ | RyR+; anti-titin+; MG symptoms NR | Steroids; IVIG | None reported | Died 52 days after MG onset due to respiratory failure and pulmonary infection |
| P043 | Pablo Sanchez-Sancho, 2021 | Spain | 63 | Male | Undifferentiated liposarcoma | Pembrolizumab | Chemotherapy | 6 | NR | NR | Tn↑; ECG: complete bundle branch block; Echo+ | Myalgia/proximal weakness; CK↑; EMG+; muscle biopsy+ | Diplopia/ptosis/dysphagia/dysphonia; AChR+; NMJ testing− | Pulse steroids; IVIG; MMF | Pacemaker; high-flow oxygen | Improved and discharged after 35 days |
| P044 | Antonio Portolés Hernández, 2021 | Spain | 48 | Female | Thymoma | Pembrolizumab | None | 10 | 3 days | None | Dyspnea; Tn↑; ECG: complete AV block; CAG−; Echo+; EMB+ | CK↑ | AChR+ | Steroids; IFX | Pacemaker | Died 10 days after admission due to heart failure |
| P045 | Sawyer J. Bawek, 2021 | USA | 68 | Male | Melanoma; metastatic neuroendocrine carcinoma | Nivolumab | None reported | 4 | NR | NR | Dyspnea/syncope; Tn↑; ECG: LBBB/third-degree AV block; CAG−; Echo− | Muscle enzyme elevation reported | Diplopia/dysphagia/ptosis; MG Ab− | Steroids; IVIG; PYR | Pacemaker; mechanical ventilation | Died due to multiple organ failure |
| P046 | Yuchen Yang, 2021 | China | 66 | Female | Right-sided colon cancer | Sintilimab | Regorafenib | 15 | NR | Hypertension; diabetes mellitus | Dyspnea; Tn↑; ECG: PACs/QT prolongation/ST-T and Q-wave abnormalities; Echo+ | Fatigue/muscle weakness; CK↑ | MG/NMJ details NR | Steroids; IVIG | None reported | Died due to tumor progression |
| P047-1 | Jessica D. Jenkins, 2021 | Canada | 71 | Male | Melanoma | Pembrolizumab | None reported | 5 | NR | NR | Tn↑; ECG: transient LBBB; Echo−; CMR− | Dysphagia/weakness; CK↑; EMG+; muscle biopsy+ | Ptosis/diplopia/dysarthria/dysphagia; AChR−; MuSK− | Steroids; IVIG; PYR | NR | Improved; discharged after 6 days |
| P047-2 | Jessica D. Jenkins, 2021 | Canada | 65 | Female | Metastatic squamous cell carcinoma | Pembrolizumab | None reported | 8 | NR | NR | Tn↑; ECG−; Echo−; CMR+ | Myalgia/proximal weakness; CK↑; EMG+ | Dysphonia/dysphagia/dysarthria; AChR−; MuSK−; RNS− | Steroids; IVIG | NR | NR |
| P048 | Jesús Fuentes-Antrás, 2020 | Spain | 75 | Male | Lung adenocarcinoma | Pembrolizumab | None | 21 | 7 days | None | Palpitations; Tn↑; ECG: complete AV block; EMB/pathology+ | Severe fatigue/myalgia; CK↑; muscle pathology+ | AChR+; PYR used | Pulse steroids; IVIG; IFX; PYR | Pacemaker | Died after 7 days of hospitalization |
| P049 | Shengnan Wang, 2021 | China | 65 | Male | Recurrent colon cancer after surgery | Tislelizumab | Chemotherapy | 20 | 15 days | None | Palpitations; Tn↑; Echo− | Limb weakness; CK↑; EMG− | Ptosis; neostigmine test+; anti-titin+ | Pulse steroids; IVIG | Mechanical ventilation reported | Improved and discharged after 23 days; weaned from non-invasive ventilation after 3 months |
| P050 | Hardeep S. Ahdi, 2023 | USA | 58 | Male | Renal cell carcinoma | Pembrolizumab | None | 25 | 4 days | Non-alcoholic steatohepatitis | Tn↑; Echo− | Diffuse myalgia/weakness; CK↑; EMG+ | Ptosis; AChR−; MuSK−; PYR used | Steroids; IVIG; PYR | NR | Recovered and discharged after 1 month |
| P051 | Divya Shah, 2023 | USA | 74 | Male | Bladder squamous cell carcinoma | Pembrolizumab | None reported | 7 | 10 days | Hypertension; hyperlipidemia | Tn↑; ECG: complete heart block; Echo+ | Proximal pain/weakness; CK↑ | Ptosis/ophthalmoplegia; AChR−; VGCC−; striational Ab+ | Pulse steroids; IVIG; MMF; PLEX | Pacemaker | Died 14 days after admission due to hypotension and pulseless electrical activity |
| P052 | Xinqing Lin, 2023 | China | 51 | Male | Left lung squamous cell carcinoma | Sintilimab | Chemotherapy | 19 | NR | Hypertension | Dyspnea/chest tightness; Tn↑; ECG: sinus tachycardia/PVCs/T-wave changes; Echo+ | Fatigue/myalgia; CK↑; EMG+; muscle biopsy+ | Ptosis; RyR-Ab reported; PYR used | Steroids; IVIG; PYR | Mechanical ventilation reported | Improved and discharged on day 18; recurrence after ICI rechallenge |
| P053 | Sean Zadeh, 2024 | USA | 80 | Male | Metastatic bladder cancer | Pembrolizumab | None | 14 | 7 days | NR | Dyspnea/orthopnea; Tn↑; Echo−; EMB+ | Myalgia/limb weakness; CK↑; EMG+ | Hoarseness/hypophonia/diplopia/dysarthria; AChR+ | Pulse steroids; PLEX; eculizumab | NR | Improved and discharged after 107 days; died 8 months later due to tumor progression-related infection |
| P054 | Stefano H. Byer, 2024 | USA | 81 | Female | Melanoma | Nivolumab | None | 20 | NR | NR | Dyspnea; Tn↑; ECG: sinus tachycardia; Echo− | Fatigue/myalgia; CK↑ | Ptosis/dysphagia; AChR+; NIF reduced | Steroids; IVIG; ABA; ruxolitinib; PYR | Mechanical ventilation reported | Survived |
| P055-1 | Nikhil Aggarwal, 2024 | United Kingdom | 58 | Male | Melanoma | Nivolumab plus ipilimumab | None | 10 | NR | Hypertension; depression; prior stereotactic radiotherapy | Palpitations/chest pain; Tn↑; ECG: RBBB/complete heart block; EMB+ | CK↑ | Diplopia; bulbar/respiratory involvement reported | Pulse steroids; IVIG; MMF; PLEX; RTX | Pacemaker; non-invasive ventilation | Discharged after 3 months |
| P055-2 | Nikhil Aggarwal, 2024 | United Kingdom | 78 | Male | Bladder cancer | NR | None | NR | NR | None | Tn↑; ECG− | Dropped head/fatigue; CK↑ | Diplopia; AChR+ | Pulse steroids; IVIG; PYR; MMF | Non-invasive ventilation | Recovered and discharged after 6 weeks |
| P055-3 | Nikhil Aggarwal, 2024 | United Kingdom | 77 | Male | Clear cell renal cell carcinoma | Nivolumab | None | NR | NR | Chronic pancreatitis; hypertension | Tn↑; ECG: dynamic T-wave inversion/ST depression | Dysphagia/dysphonia; CK↑ | Ptosis; AChR− | Steroids; IVIG; PYR | Non-invasive ventilation | Died 3 weeks after admission due to respiratory failure |
| P055-4 | Nikhil Aggarwal, 2024 | United Kingdom | 86 | Male | Melanoma | Pembrolizumab | None | 14 | NR | Hypertension; type 2 diabetes mellitus; osteoporosis; cervical spondylosis; prostate cancer | Tn↑; ECG: complete AV block/LBBB/long pauses | Fatigue; CK↑ | Diplopia/slurred speech; AChR+ | Steroids; IVIG; neostigmine | Pacemaker | Died 2 days after admission due to respiratory failure |
| P056 | Takahiro Yanase, 2021 | Japan | 59 | Male | Metastatic renal cell carcinoma | Ipilimumab plus nivolumab | None | 21 | NR | None | Tn↑; ECG: complete AV block; CAG− | CK↑ | Ptosis/ocular movement impairment; AChR+ | Pulse steroids; PLEX; IVIG | NR | Improved after 6 months |
| P057 | Sanziana R. I. Schiopu, 2021 | Germany | 75 | Male | Metastatic malignant mesothelioma | Pembrolizumab | None reported | NR | At presentation | None | Dyspnea; Tn↑; ECG: ST elevation/PVCs; CAG−; Echo+ | Generalized weakness; CK↑ | Diplopia/ptosis; AChR−; MuSK− | Steroids; PLEX | Mechanical ventilation reported | Improved after 1 month |
| P058 | Jason Cham, 2021 | USA | 72 | Male | Prostate cancer | Durvalumab | None reported | 18 | 4 days | Prostate cancer history; central serous retinopathy; obstructive sleep apnea; type 2 diabetes mellitus; hypertension; mild COPD | Dyspnea/chest tightness; Tn↑; ECG: ST elevation; CAG−; Echo− | Weakness; CK↑ | Dysphagia; AChR binding/blocking Ab+ | Steroids; PLEX | Mechanical ventilation reported | Unable to wean from ventilation after 36 days |
| P059 | Fadi Nasr, 2018 | France | 79 | Male | Metastatic gastric adenocarcinoma | Pembrolizumab | None reported | 35 | 10 days | NR | Tn↑; ECG: progressed to third-degree AV block; Echo− | Ptosis/weakness; CK↑; ocular MRI+; muscle biopsy+ | Asymmetric ptosis/ataxia; AChR−; MuSK−; RNS− | Prednisone/steroids; PYR; PLEX; IVIG; MTX | Pacemaker | Transferred to oncology department for rehabilitation |
| P060 | T. Shirai, 2019 | Japan | 83 | Male | Metastatic melanoma | Pembrolizumab | None reported | 25 | 4 days | NR | Tn↑; ECG: T-wave abnormality/wide QRS/first-degree AV block | Neck weakness/myalgia; CK↑; muscle MRI+ | Ptosis/diplopia/dysphagia; AChR+; striational Ab+; RNS− | Pulse steroids; PLEX | NR | Improved after 6 weeks |
| P061 | Mahdieh Fazel, 2019 | USA | 78 | Female | Metastatic melanoma | Nivolumab plus ipilimumab | None reported | 5 | NR | Hypertension; intermittent asthma; prior pulmonary embolism; depression | Cardiac details limited; Echo− | Proximal weakness/myalgia; CK↑; muscle MRI+; myositis Ab− | Diplopia/gaze palsy; MG/NMJ serology details NR | Steroids; IVIG; PLEX | Mechanical ventilation reported | Died due to respiratory failure |
| P062 | Hayato So, 2019 | Japan | 55 | Female | Metastatic melanoma | Nivolumab | None reported | 14 | NR | Thymoma | Dyspnea; Tn↑; ECG: wide QRS tachycardia; Echo+ | Neck stiffness/myalgia/limb weakness; CK↑; EMG+; muscle MRI+; muscle biopsy+ | Ophthalmoplegia/ptosis/dysphagia; AChR+; RNS−; SFEMG+ | Steroids; IVIG; PLEX | Mechanical ventilation reported | Able to walk after 180 days |
| P063 | Smathorn Thakolwiboon, 2019 | USA | 87 | Male | Bladder urothelial carcinoma | Atezolizumab | None reported | 21 | NR | Hypertension; dyslipidemia; chronic kidney disease | ECG: new RBBB/LAFB; Tn details NR | Proximal weakness; CK↑; myositis Ab− | Diplopia/ptosis/nasal voice; striational Ab+; AChR blocking Ab+; MuSK−; VGCC− | Prednisone; IVIG; PLEX; PYR | NR | Died due to cardiac arrest |
| P064 | Justin B. Hellman, 2019 | USA | 84 | Male | High-grade urothelial carcinoma | Pembrolizumab | None reported | 4 | NR | NR | Tn↑; ECG: conduction abnormality/complete heart block; CAG−; Echo− | Gait difficulty/generalized weakness; CK↑; EMG+ | Ptosis/dysphagia; AChR−; NMJ abnormality not shown | Prednisone | Pacemaker; mechanical ventilation reported | Died 33 days after cardiac arrest/resuscitation |
| P066 | Maki Todo, 2020 | Japan | 63 | Male | Bladder cancer | Pembrolizumab | None reported | 34 | 12-34 days | NR | Tn↑; ECG: RBBB; Echo− | CK↑; EMG− | Ptosis/diplopia; AChR−; MuSK−; RNS−; Tensilon− | Prednisolone | NR | Improved; corticosteroids discontinued after 362 days |
| P067 | Toshihiro Kimura, 2016 | Japan | 80 | Male | Metastatic melanoma | Nivolumab | None reported | 17 | NR | NR | Severe dyspnea; Tn↑; Echo+; CAG− | Muscle weakness; CK↑; muscle biopsy+ | AChR+; PYR used | Pulse steroids; immunoadsorption; PLEX; IVIG; PYR | Mechanical ventilation reported | Improved after 4 months |
| P068 | Arjun Basnet, 2024 | USA | 76 | Male | Melanoma | Ipilimumab plus nivolumab | None reported | 21 | 4 days | Coronary artery disease; hypertension; hyperlipidemia; diabetes mellitus; hyperthyroidism | Progressive dyspnea; Tn↑; ECG: ventricular arrhythmia; CAG+; Echo− | Diffuse weakness; CK↑ | Facial/bulbar/respiratory weakness; MG-specific Ab+ | Steroids | CRRT; mechanical ventilation reported | Died on hospital day 13 due to cardiac arrest |
| P069 | Christopher Nelke, 2024 | Germany | 47 | Female | Breast cancer; pre-existing thymoma-associated myasthenia gravis | Pembrolizumab | None reported | 84 | NR | Pre-existing thymoma-associated myasthenia gravis | Myocarditis; Tn↑; CMR+ | Myalgia; CK↑; EMG+; muscle MRI+; muscle biopsy+ | Pre-existing MG; current NMJ details NR | Steroids; immunoadsorption; eculizumab | NR | Improved; discharged |
| P070-1 | Derrick Ho Wai Siu, 2022 | Australia | 73 | Male | Lung cancer | Pembrolizumab | Carboplatin; paclitaxel | 33 | NR | Hypertension; atrial fibrillation; resected early-stage colorectal cancer; benign prostatic hyperplasia | Dyspnea; series-level Tn/ECG abnormalities reported; individual details NR | Neck flexion weakness; series-level CK↑ reported | Dysphagia; hypophonia; neck weakness | Steroids; IVIG | NR | Improved; discharged after 41 days |
| P070-2 | Derrick Ho Wai Siu, 2022 | Australia | 74 | Male | Urothelial bladder cancer | Avelumab | None reported | 21 | NR | Hypertension; obstructive sleep apnea; COPD; hypothyroidism; gout; history of prostate cancer treated with radical prostatectomy | Series-level myocarditis evidence reported; individual details NR | Proximal leg myalgia/weakness; series-level CK↑ reported | Fatigable diplopia | Steroids; MMF | NR | Improved after 34 days |
| P070-3 | Derrick Ho Wai Siu, 2022 | Australia | 67 | Female | Lung adenocarcinoma | Atezolizumab | None reported | 63 | NR | Hypertension; hypercholesterolemia; type 2 diabetes mellitus; gout | Dyspnea; series-level Tn/ECG abnormalities reported; individual details NR | Fatigue; series-level CK↑ reported | Diplopia/ptosis | Prednisone | NR | Died after 14 days |
| P070-4 | Derrick Ho Wai Siu, 2022 | Australia | 81 | Male | Melanoma | Pembrolizumab | None reported | 28 | NR | Hypertension; localized prostate cancer; osteoarthritis | Exertional dyspnea; series-level myocarditis evidence reported; individual details NR | Fatigue; series-level CK↑ reported | Binocular diplopia | Prednisone; IVIG | NR | Died after 10 days |
| P070-5 | Derrick Ho Wai Siu, 2022 | Australia | 74 | Male | Melanoma | Nivolumab | None reported | 56 | NR | Hypertension; hypercholesterolemia; atrial fibrillation; history of prostate cancer treated with radical prostatectomy; idiopathic peripheral neuropathy | Exertional dyspnea; series-level Tn/ECG abnormalities reported; individual details NR | Muscle weakness; series-level CK↑ reported | Hoarseness/dysphagia | Steroids; IVIG; MMF | NR | Improved after 32 days |
| P070-6 | Derrick Ho Wai Siu, 2022 | Australia | 63 | Male | Renal cell carcinoma | Pembrolizumab | None reported | 18 | NR | Hypertension; hypercholesterolemia; type 2 diabetes mellitus; CKD stage III; nonobstructive CAD | Chest pain/dyspnea; series-level Tn/ECG abnormalities reported; individual details NR | Fatigue; series-level CK↑ reported | MG/NMJ symptoms NR | Steroids; MMF; IVIG | NR | Improved after 20 days |
| P070-7 | Derrick Ho Wai Siu, 2022 | Australia | 73 | Male | Renal cell carcinoma | Nivolumab plus ipilimumab | None reported | 25 | NR | Resected meningioma; early breast cancer; previous venous thromboembolism | Exertional dyspnea; series-level myocarditis evidence reported; individual details NR | Neck, upper- and lower-limb weakness; series-level CK↑ reported | Dysphonia/ptosis/diplopia | Steroids; PLEX; IVIG; MMF | NR | Improved; discharged after 61 days |
| P071-1 | Jamie MJ Weaver, 2023 | United Kingdom | 56 | Male | Renal cancer | Nivolumab plus ipilimumab | None reported | After cycle 1 | NR | NR | Myocarditis evidence limited; Tn not available; Echo not done | CK↑ | Diplopia/ptosis; AChR+ | Steroids; IVIG; MMF; PYR | Nasal oxygen | Discharged after 5 days; died at 7 months due to cancer progression/relapsed MG |
| P071-2 | Jamie MJ Weaver, 2023 | United Kingdom | 45 | Male | Melanoma | Nivolumab plus ipilimumab | None reported | After cycle 2 | NR | NR | Tn↑; Echo− | CK not elevated | Diplopia/ptosis; anti-Ro52+ | Steroids; MMF; PYR | None required | Discharged after 4 days; died at 3 months due to cancer progression |
| P071-3 | Jamie MJ Weaver, 2023 | United Kingdom | 58 | Male | Melanoma | Nivolumab plus ipilimumab | None reported | After cycle 1 | NR | NR | Tn↑; conduction disease/trifascicular block; Echo− | Neck weakness; CK↑ | Diplopia/ptosis/nasal speech/dysphagia; MuSK+ | Steroids; IVIG; MMF; PYR; ABA; RTX | CPAP | Discharged after 63 days; died at 8 months due to cancer progression |
| P071-4 | Jamie MJ Weaver, 2023 | United Kingdom | 73 | Male | Melanoma | Nivolumab plus ipilimumab | None reported | After cycle 1 | NR | NR | Tn↑; complete heart block; Echo− | Myalgia; CK↑ | Diplopia/ptosis; AChR+; anti-Ro52+ | Steroids; IVIG; MMF; PYR; ABA; RTX; PLEX | IPPV; pacing for complete heart block | Died as inpatient from MG after 53 days |
| P071-5 | Jamie MJ Weaver, 2023 | United Kingdom | 76 | Male | Melanoma | Pembrolizumab | None reported | After cycle 2 | NR | NR | Dyspnea; Tn↑; Echo− | Myalgia; CK↑ | Diplopia/ptosis; AChR+ | Steroids; IVIG; MMF; PYR | None required | Discharged after 10 days; died at 8 months unrelated to cancer or MG |
| P071-6 | Jamie MJ Weaver, 2023 | United Kingdom | 80 | Male | Melanoma | Pembrolizumab | None reported | After cycle 2 | NR | NR | Tn↑; Echo+ | CK↑ | Diplopia/ptosis; anti-Ro52+ | Steroids; IVIG; MMF; PYR | None required | Improved; discharged after 7 days; alive at 1 year |
| P071-7 | Jamie MJ Weaver, 2023 | United Kingdom | 75 | Male | Renal cancer | Nivolumab | None reported | After cycle 2 | NR | NR | Tn↑; Echo+ severe LVSD, previous MI | CK↑ | Diplopia/ptosis; autoantibodies negative | Steroids; IVIG; MMF; PYR | None required | Improved; discharged after 5 days; alive at 1 year |
| P071-8 | Jamie MJ Weaver, 2023 | United Kingdom | 82 | Female | Colorectal cancer | Pembrolizumab | None reported | After cycle 2 | NR | NR | Tn↑; Echo− | Myalgia; CK↑ | Diplopia/ptosis; AChR+ | Steroids; IVIG; MMF; PYR | Nasal oxygen | Improved; discharged after 7 days; alive at 1 year |
| P071-9 | Jamie MJ Weaver, 2023 | United Kingdom | 86 | Male | Melanoma | Nivolumab | None reported | After cycle 2 | NR | NR | Tn↑; Echo− | Myalgia; CK↑ | Diplopia/ptosis; AChR+ | Steroids; IVIG; MMF; PYR | HFNS | Improved; discharged after 74 days; alive at 1 year |
| P071-10 | Jamie MJ Weaver, 2023 | United Kingdom | 76 | Male | Bladder cancer | Atezolizumab | None reported | After cycle 1 | NR | NR | Myocarditis evidence limited; Tn not available; Echo not done | CK↑ | Diplopia/ptosis; AChR+ | Prednisolone; MMF; PYR | None required | Improved; discharged after 2 days; alive at 1 year |
| P072 | Zhang Yu, 2023 | China | 64 | Female | Urothelial carcinoma | NR | Chemotherapy | 20 | NR | NR | Tn↑; Echo+; myocardial perfusion imaging+; CMR+; CAG− | CK↑; EMG+ | Ptosis; RNS− | Steroids | None reported | NR |
| P073 | Zeng Chenxin, 2024 | China | 64 | Female | NR | Sintilimab | NR | 30 | NR | Hypertension | Chest tightness; Tn↑; ECG: QT prolongation/ischemic changes/LAFB; CAG−; CMR+ | Diffuse weakness; CK↑; EMG+; muscle MRI− | Diplopia/ptosis | Steroids; IVIG | None reported | NR |
| P074 | Han Le, 2024 | China | 78 | Male | NR | NR | NR | 44 | NR | Hypertension; diabetes mellitus | Chest tightness; Tn↑; ECG+; Echo−; CAG+ | Neck/back fatigue; CK↑ | Ptosis; choking with water/food; hoarseness; Jolly test+; AChR−; MuSK−; RNS− | Steroids; PLEX | None reported | NR |
| P075 | Nannan Fan, 2021 | China | 59 | Male | Hepatocellular carcinoma | NR | Regorafenib | 18 | NR | None | Tn↑; ECG−; Echo− | Diffuse weakness; myalgia; CK↑ | Ptosis | Steroids; IVIG; RTX | None reported | NR |
| P077 | Maia Ioana Mihon, 2025 | Romania | 83 | Male | Melanoma | Pembrolizumab | None | 30 | NR | NR | Syncope; Tn↑; ECG: Mobitz II AV block/RBBB; Echo− | Proximal weakness; CK↑ | Dysphonia/dysarthria/ptosis/dysphagia; myositis/MG Ab−; neostigmine test− | Pulse steroids | Pacemaker | Died within 10 days due to respiratory failure |
| P078 | Ben Schroeder, 2025 | USA | 70 | Female | Invasive bladder cancer | Pembrolizumab | None reported | 21 | NR | NR | Tn↑; ECG−; Echo−; CMR+ | Myalgia/weakness; CK↑; muscle MRI+ | Ptosis; MG Ab−; NIF reduced | Steroids; PLEX | NR | Improved and discharged after 1 month following readmission |
| P079 | Baheti Kalifu, 2025 | China | 68 | Male | Primary liver cancer | Camrelizumab | Lenvatinib | 17 | 10 days | NR | Tn↑; ECG: sinus tachycardia/ST-T changes; Echo+ | Generalized weakness; CK↑; EMG/myogenic injury suspected | Ptosis/choking; anti-titin Ab strongly positive | Steroids | NR | Recovered on 1-month follow-up |
| P080 | Daniela Garcez, 2022 | Portugal | 83 | Female | Hepatocellular carcinoma | Pembrolizumab | None reported | 15 | 5 days | None | Tn↑; ECG: LBBB; Echo− | Fatigue; CK↑ | Blurred vision/ptosis/diplopia; PYR used | Pulse steroids; IVIG; PYR | NR | Mostly improved after 6 months |
| P081-1 | Umar Ismail, 2025 | United Kingdom | 67 | Male | Melanoma | Ipilimumab plus nivolumab | None | 7 | NR | NR | Dyspnea; Tn↑; ECG−; Echo−; CMR+ | Myalgia/weakness; CK↑ | AChR−; MG/NMJ details limited | Steroids; MMF | NR | Improved and discharged after 15 days |
| P081-2 | Umar Ismail, 2025 | United Kingdom | 84 | Male | Melanoma | Pembrolizumab | None | 14 | NR | History of prostate cancer | Dyspnea; Tn↑; ECG: RBBB; Echo+ | Severe fatigue/weakness; CK↑ | Diplopia/ptosis/ophthalmoplegia; AChR− | Pulse steroids; IVIG; MMF; TCZ; TAC; PYR | None reported | Died after 15 days |
| P081-3 | Umar Ismail, 2025 | United Kingdom | 68 | Male | Esophageal cancer and lung cancer | Pembrolizumab | None reported | 28 | 11 days | NR | Tn↑; ECG−; Echo− | Fatigue; CK↑ | Ptosis/slurred speech/bulbar symptoms; antibody details NR | Prednisone; IVIG; TAC | None reported | Died after 7 days |
| P081-4 | Umar Ismail, 2025 | United Kingdom | 60 | Male | Clear cell renal cell carcinoma | Ipilimumab plus nivolumab | None reported | 14 | NR | Obesity; chronic kidney disease stage 1; atrial fibrillation | Dyspnea; Tn↑; ECG: sinus tachycardia; CMR+ | Fatigue; CK↑; muscle MRI+ | Dysphagia; AChR−; MuSK− | Steroids; IVIG; MMF; PYR | None reported | Improved and discharged after 22 days |
| P082 | Sean Halloran, 2025 | USA | 59 | Male | Hepatocellular carcinoma | Tremelimumab plus durvalumab | None reported | 19 | 3 days | NR | Tn↑; ECG−; CAG−; Echo: abnormal GLS; CMR−; EMB+ | CK↑ | Ophthalmoplegia/ptosis; MG Ab− | Steroids; ABA; ruxolitinib; IVIG; PYR | None reported | Improved and discharged after 27 days |
| P084 | Andrea Rigamonti, 2025 | Italy | 71 | NR | NR | NR | NR | NR | NR | Pre-existing ocular myasthenia gravis | Myocarditis evidence NR | EMG+ | MG symptoms; RNS+ | PYR; prednisone; PLEX; IVIG; eculizumab | NR | NR |
| P085 | Khaled M. Darwesh, 2025 | USA | 75 | Male | Lung cancer | Pembrolizumab | None reported | 28 | NR | NR | Dyspnea; Tn↑; ECG: first-degree AV block; Echo− | Lower-limb weakness/thigh pain; CK↑ | Diplopia/ptosis/dysphagia/weak voice; AChR+; NIF reduced | Steroids; IVIG | Mechanical ventilation reported | Died after 13 days |
| P086 | Jeanne Emile, 2024 | France | 72 | Male | Melanoma | Nivolumab | None reported | 28 | NR | Pre-existing ocular myasthenia gravis | Dyspnea; Tn↑; ECG: LBBB/sinus tachycardia; CAG−; Echo+; EMB+ | CK↑ | Diplopia/ptosis/dysphagia | Steroids; IVIG; PLEX; anakinra; PYR | Mechanical ventilation reported | Died after 3 months due to respiratory failure |
| P087 | Lucía Gómez, 2025 | Spain | 80 | Male | Gastric adenocarcinoma | Pembrolizumab | None reported | 21 | NR | Hypertension; hypercholesterolemia; chronic ischemic heart disease | Tn↑; ECG: high-grade AV block; Echo+; CMR+ | Fatigue/weakness; CK↑; EMG+ | Diplopia/ptosis/blurred vision; anti-titin+ | Steroids; IVIG; IFX | Pacemaker | Improved and discharged after 1 month; died 3 weeks after discharge due to general deterioration |
| P088 | Mingjia Lu, 2025 | China | 69 | Female | Bladder cancer | Tislelizumab | None reported | 16 | NR | Pre-existing MG; hypertension; primary aldosteronism; type 2 diabetes mellitus | Chest tightness/dyspnea/orthopnea; Tn↑; ECG−; Echo+; CMR+ | Neck weakness/fatigue; CK↑ | Ptosis/diplopia/dysarthria; pre-existing MG | Steroids; efgartigimod; prednisone | None reported | Improved after 1 month |
| P089 | Ferdinand Otto, 2024 | Austria | 75 | Female | Melanoma | Nivolumab plus ipilimumab | None reported | 21 | NR | Hypertension; chronic immune thyroiditis | Fatigue; Tn↑; ECG−; Echo− | Ascending muscle weakness; CK↑ | Diplopia/gaze palsy; AChR+; striational Ab+ | Pulse steroids; IVIG; RTX | NR | Died after 46 days |
| P090-1 | Benjamin Jinsung Park, 2025 | USA | 62 | Male | Mesothelioma | Nivolumab plus ipilimumab | Cabozantinib | 14 | NR | Chronic myeloid leukemia | Dyspnea; Tn↑ | Generalized/limb weakness; CK↑ | Diplopia/ptosis; AChR− | Steroids; IVIG; PLEX | None reported | Improved and discharged after 26 days |
| P090-2 | Benjamin Jinsung Park, 2025 | USA | 76 | Female | Lung cancer | Pembrolizumab | None reported | 7 | NR | NR | Tn↑; ECG: VT | Generalized/proximal weakness; CK markedly↑; orbital MRI+ | Ptosis/dysphagia/hypophonia; MG Ab− | Pulse steroids; IVIG; MMF; PLEX; PYR | Mechanical ventilation reported | Died after 5 days |
| P091 | Stephanie S. Cobelas-Cartagena, 2025 | Spain | 72 | Male | Melanoma | Pembrolizumab | None reported | 15 | NR | Type 2 diabetes mellitus | Tn↑; ECG: RBBB/AF/flutter/complete AV block; Echo− | Lower-limb weakness/myalgia; CK↑; EMG+ | Ptosis/ophthalmoplegia/dysphagia; AChR+; anti-titin+ | Steroids; TAC; PLEX; IVIG; cyclosporine; efgartigimod; PYR | Pacemaker; mechanical ventilation | Extubated after 40 days; discharged after 70 days |
| P092 | Ching-Cheng Chan, 2026 | China | 79 | Male | Urothelial carcinoma | Pembrolizumab | None reported | 26 | 6 days | Coronary artery disease; aortic valve regurgitation status post surgical replacement | Chest tightness; Tn↑; ECG: third-degree AV block/RBBB; CAG−; Echo− | Neck/proximal weakness; CK↑; EMG+ | Ptosis/dysphagia/dysphonia/dysarthria; AChR+; SFEMG+ | Steroids; IVIG; MMF; PLEX; PYR | Pacemaker; mechanical ventilation | Improved and weaned from ventilation after 83 days |
| P093 | Yasmin Abozenah, 2025 | USA | 75 | Female | Endometrial cancer | Dostarlimab | Chemotherapy | 81 | NR | Hypertension; hypothyroidism; morbid obesity | Tn/CMR evidence limited; ECG: intermittent bilateral bundle branch block; Echo−; CMR+ | Generalized muscle weakness; CK mildly↑ | Severe dysphagia/ptosis/diplopia/hoarseness; AChR+ | Pulse steroids; MMF; ABA; IVIG; tofacitinib | Mechanical ventilation reported | Improved |
| P094 | Eman Albastaki, 2025 | United Kingdom | 80 | Male | Lung cancer | Pembrolizumab | None reported | 28 | 7 days | Chronic kidney disease; renal cell carcinoma; type 2 diabetes mellitus; hypertension | Dyspnea; Tn↑; ECG−; Echo− | Neck flexion weakness; CK↑ | Ptosis/dysarthria/dysphagia; neostigmine used | Steroids; IVIG; neostigmine | Mechanical ventilation reported | Died after 6 days |
| P095 | Jad Daw, 2025 | USA | 79 | Male | Melanoma | Nivolumab plus ipilimumab | None reported | 21 | NR | Mitral valve prolapse status post mitral valve repair; adrenal insufficiency; hypothyroidism | Fatigue; Tn↑; ECG: junctional tachycardia/RBBB; Echo−; CMR+ | CK↑ | MG/NMJ details NR | Prednisone | None reported | Improved |
| P096 | Omar Fakhreddine, 2025 | USA | 86 | Male | Clear cell renal cell carcinoma | Pembrolizumab | Axitinib | 20 | NR | Dyslipidemia; benign prostatic hyperplasia | Dyspnea; Tn↑; ECG: AV block progressing to complete heart block; Echo− | Neck weakness; CK↑ | Diplopia/ptosis/ophthalmoplegia/dysphagia; AChR+ | Steroids | Pacemaker | Died after 28 days |
| P097 | Ziad Gaafar, 2026 | United Kingdom | 74 | Male | Clear cell renal cell carcinoma | Pembrolizumab | None reported | 7 | 2 days | Hypertension | Tn↑; ECG: irregular rhythm/R-wave progression abnormality/T-wave inversion; Echo− | Neck stiffness/weakness/back pain; CK↑; EMG/SFEMG+ | Ptosis/neck flexor weakness; PYR used | Pulse steroids; IVIG; PYR | NR | Improved |
| P098 | Lingling Zhao, 2025 | China | 53 | Male | Thymoma | Camrelizumab | None reported | 14 | 1 day | NR | Palpitations/wheezing; Tn↑; ECG: sinus tachycardia; Echo− | Fatigue; CK↑ | Diplopia/ptosis; AChR+ | Pulse steroids; IVIG; PYR | NR | Improved |

NOTE:1.Abbreviations: ABA, abatacept; AChR, acetylcholine receptor antibody; AF, atrial fibrillation; AV, atrioventricular; AZA, azathioprine; CAG, coronary angiography; CK, creatine kinase; CMR, cardiac magnetic resonance; COPD, chronic obstructive pulmonary disease; CRRT, continuous renal replacement therapy; ECG, electrocardiography; Echo, echocardiography; EMB, endomyocardial biopsy; EMG, electromyography; GLS, global longitudinal strain; HFNS, high-flow nasal support; ICI, immune checkpoint inhibitor; IFX, infliximab; IPPV, invasive positive-pressure ventilation; irAE, immune-related adverse event; IVIG, intravenous immunoglobulin; LAFB, left anterior fascicular block; LBBB, left bundle branch block; LVSD, left ventricular systolic dysfunction; MG, myasthenia gravis; MMF, mycophenolate mofetil; MRI, magnetic resonance imaging; MTX, methotrexate; NCS, nerve conduction study; NIF, negative inspiratory force; NMJ, neuromuscular junction; NR, not reported; NSTEMI, non-ST-segment elevation myocardial infarction; NT-proBNP, N-terminal pro-B-type natriuretic peptide; PAC, premature atrial contraction; PLEX, plasma exchange; PVC, premature ventricular contraction; PYR, pyridostigmine; RBBB, right bundle branch block; RNS, repetitive nerve stimulation; RTX, rituximab; RyR, ryanodine receptor; SFEMG, single-fiber electromyography; SVPC, supraventricular premature contraction; TAC, tacrolimus; TCZ, tocilizumab; Tn, troponin; VGCC, voltage-gated calcium channel; VT, ventricular tachycardia.

2.A plus sign (+) indicates an abnormal or supportive finding; a minus sign (−) indicates a reported negative or normal finding; NR indicates not reported. Diagnostic evidence was extracted as reported in the original case reports or case series. Absence of a finding in this table indicates that it was not reported, unless explicitly stated as negative or normal.

3.For case series in which laboratory, ECG, echocardiographic, CMR, EMG/NCS, or biopsy findings were summarized at the cohort level but not mapped to individual patients, the corresponding patient-level cells were recorded as “series-level evidence reported; individual details NR.”

4.Time from ICI initiation to symptom onset and time from presentation to irAE-directed treatment are reported in days when available; text entries such as “after cycle 1,” “at presentation,” or “within 24 hours” were retained when exact day counts were not reported. Supportive/cardiac interventions include respiratory support, pacemaker or ICD placement, CRRT, and other explicitly reported supportive measures. Pacemaker or ICD placement was not considered mechanical circulatory support.
