# Supplementary material for: Immune checkpoint inhibitor–related myositis, myocarditis, and myasthenia gravis overlap syndrome: a systematic review and pooled analysis of individual cases
Source: Front Immunol. 2026 Jul 29;17:1854723. doi: 10.3389/fimmu.2026.1854723 (PMC13461781; doi:10.3389/fimmu.2026.1854723)
Supplement: Supplementary file 3 [file Table2.docx]

| **Category** | **Variable** | **n/N among patients with available information (%)** | **n/total cohort (%)** |
| --- | --- | --- | --- |
| Reported comorbidities | Comorbidity information available | 79/136 (58.1) | 79/136 (58.1) |
| Reported comorbidities | Comorbidity information not reported | 57/136 (41.9) | 57/136 (41.9) |
| Reported comorbidities | No reported comorbidity | 13/79 (16.5) | 13/136 (9.6) |
| Reported comorbidities | At least one reported comorbidity or relevant medical history | 66/79 (83.5) | 66/136 (48.5) |
| Reported comorbidities | Hypertension | 43/79 (54.4) | 43/136 (31.6) |
| Reported comorbidities | Diabetes mellitus | 20/79 (25.3) | 20/136 (14.7) |
| Reported comorbidities | Chronic kidney disease | 9/79 (11.4) | 9/136 (6.6) |
| Reported comorbidities | Coronary artery disease or ischemic heart disease | 9/79 (11.4) | 9/136 (6.6) |
| Reported comorbidities | Obesity | 3/79 (3.8) | 3/136 (2.2) |
| Reported comorbidities | Dyslipidemia, hyperlipidemia, or hypercholesterolemia | 17/79 (21.5) | 17/136 (12.5) |
| Reported comorbidities | Thyroid disorder or thyroid disease history | 10/79 (12.7) | 10/136 (7.4) |
| Reported comorbidities | Atrial fibrillation | 6/79 (7.6) | 6/136 (4.4) |
| Reported comorbidities | Pre-existing myasthenia gravis | 4/79 (5.1) | 4/136 (2.9) |
| Reported comorbidities | Chronic pulmonary disease, COPD, or asthma | 4/79 (5.1) | 4/136 (2.9) |
| Reported comorbidities | Obstructive sleep apnea | 3/79 (3.8) | 3/136 (2.2) |
| Reported comorbidities | Peripheral vascular or thromboembolic disease | 3/79 (3.8) | 3/136 (2.2) |
| Concomitant non-ICI anticancer therapy | Information on concomitant non-ICI anticancer therapy available | 59/136 (43.4) | 59/136 (43.4) |
| Concomitant non-ICI anticancer therapy | Information on concomitant non-ICI anticancer therapy not reported | 77/136 (56.6) | 77/136 (56.6) |
| Concomitant non-ICI anticancer therapy | ICI only, with no concomitant non-ICI anticancer therapy | 32/59 (54.2) | 32/136 (23.5) |
| Concomitant non-ICI anticancer therapy | ICI plus chemotherapy | 12/59 (20.3) | 12/136 (8.8) |
| Concomitant non-ICI anticancer therapy | ICI plus chemoradiotherapy | 3/59 (5.1) | 3/136 (2.2) |
| Concomitant non-ICI anticancer therapy | ICI plus targeted therapy, anti-VEGF therapy, or TKI | 12/59 (20.3) | 12/136 (8.8) |

Supplementary Table 2. Descriptive summary of reported comorbidities and concomitant non-ICI anticancer therapies

NOTE:1.Abbreviations: COPD, chronic obstructive pulmonary disease; ICI, immune checkpoint inhibitor; TKI, tyrosine kinase inhibitor; VEGF, vascular endothelial growth factor.

2.Comorbidities and concomitant non-ICI anticancer therapies were extracted as reported in the original case reports, case series, or institutional records. Unreported comorbidities were not assumed to be absent. For individual comorbidities, denominators represent patients with available comorbidity information. For concomitant non-ICI anticancer therapy, denominators represent patients with available information on concomitant non-ICI anticancer therapy.

3.The “n/total cohort” column uses the full cohort of 136 patients as the denominator. The “ICI only” category includes patients receiving single-agent ICI or dual ICI therapy without concomitant non-ICI anticancer therapy. Dual ICI therapy was not counted as concomitant non-ICI anticancer therapy.

4.Concomitant non-ICI anticancer therapy categories were treated as mutually exclusive. Targeted therapy, anti-VEGF therapy, and TKI therapy included agents such as axitinib, lenvatinib, regorafenib, cabozantinib, bevacizumab, disitamab vedotin, and sacituzumab tirumotecan when explicitly reported as concomitant or part of the same treatment regimen.
