# Supplementary material for: Immune checkpoint inhibitor–related myositis, myocarditis, and myasthenia gravis overlap syndrome: a systematic review and pooled analysis of individual cases
Source: Front Immunol. 2026 Jul 29;17:1854723. doi: 10.3389/fimmu.2026.1854723 (PMC13461781; doi:10.3389/fimmu.2026.1854723)
Supplement: Supplementary file 4 [file Table3.docx]

**Supplementary Table 3. Clinical characteristics, diagnostic evidence, treatment, and outcomes of the three institutional cases**

| **Case ID** | **Age/sex** | **Baseline comorbidities** | **Cancer type and stage** | **ICI regimen** | **Concomitant non-ICI anticancer therapy** | **Number of ICI cycles before onset** | **Time from ICI initiation to symptom onset** | **Major presenting symptoms** | **Myocarditis evidence** | **Myositis evidence** | **MG/NMJ evidence** | **Time from symptom onset/presentation to irAE-directed treatment** | **Initial corticosteroid regimen** | **Second-line immunomodulatory therapy** | **MG-directed symptomatic therapy** | **Respiratory/cardiac support** | **Length of stay** | **Outcome/disposition** | **Follow-up status** |
| --- | --- | --- | --- | --- | --- | --- | --- | --- | --- | --- | --- | --- | --- | --- | --- | --- | --- | --- | --- |
| 1 | 42/Female | None | Type B2 thymoma, stage IV | Tislelizumab | Chemotherapy | 1 | 17 days | Ptosis, dysarthria, dysphagia, neck and limb weakness, chest tightness | Chest tightness; Tn↑; ECG: ventricular arrhythmia; Echo: no major abnormality | Weakness; CK↑; myositis-associated antibodies− | Ptosis, dysarthria and dysphagia; AChR+ | 1 day | Methylprednisolone 2 mg/kg/day | PLEX; TCZ; efgartigimod | PYR, discontinued because of gastrointestinal intolerance | Mechanical ventilation | 30 days | Improved | Recovered well |
| 2 | 68/Male | History of lung cancer | Bladder cancer | Toripalimab | Disitamab vedotin | 2 | 19 days | Chest tightness, dyspnea, ptosis | Chest tightness and dyspnea; Tn↑; ECG: third-degree AV block; Echo: normal | Weakness; CK↑ | Ptosis; anti-titin Ab+ | 7 days | Methylprednisolone 500 mg | MMF; IVIG; IFX; RTX | PYR, discontinued because of conduction block | Mechanical ventilation; temporary pacemaker | 35 days | Discharged against medical advice | Died of severe pneumonia and respiratory failure |
| 3 | 60/Female | Hypertension; hyperlipidemia | Breast cancer | Pembrolizumab | Sacituzumab govitecan | 1 | 30 days | Chest tightness, dyspnea, bilateral ptosis, muscle weakness | Chest tightness and dyspnea; Tn↑; ECG: ventricular arrhythmia and high-grade AV block; Echo: reduced LV wall motion with EF 48%; CAG− | Weakness with limb muscle strength grade 1; CK↑ | Bilateral ptosis; AChR− | 1 day | Methylprednisolone 2 mg/kg/day | PLEX; IVIG | PYR, discontinued because of conduction block | Mechanical ventilation; temporary pacemaker | 15 days | Transferred to a long-term care facility | Still required ventilatory support at 1-month follow-up |

NOTE:1.Abbreviations: AChR, acetylcholine receptor antibody; AV, atrioventricular; CAG, coronary angiography; CK, creatine kinase; ECG, electrocardiography; Echo, echocardiography; EF, ejection fraction; ICI, immune checkpoint inhibitor; IFX, infliximab; IVIG, intravenous immunoglobulin; LV, left ventricular; MG, myasthenia gravis; MMF, mycophenolate mofetil; NMJ, neuromuscular junction; PLEX, plasma exchange; PYR, pyridostigmine; RTX, rituximab; TCZ, tocilizumab; Tn, troponin.

2.A plus sign (+) indicates an abnormal or supportive finding; a minus sign (−) indicates a reported negative or normal finding.
